# Are we close to defining a metabolomic signature of human obesity? A systematic review of metabolomics studies

**DOI:** 10.1007/s11306-019-1553-y

**Published:** 2019-06-13

**Authors:** Oscar Daniel Rangel-Huerta, Belén Pastor-Villaescusa, Angel Gil

**Affiliations:** 1 Faculty of Medicine, Department of Nutrition, University of Oslo, Oslo, Norway; 2 Norwegian Veterinary Institute, Oslo, Norway; 3 LMU - Ludwig-Maximilians-Universität München, Division of Metabolic and Nutritional Medicine, Dr. von Hauner Children’s Hospital, University of Munich Medical Center, Munich, Germany; 4 Institute of Epidemiology, Helmholtz Zentrum München–German Research Centre for Environmental Health, Neuherberg, Germany; 5 Department of Biochemistry and Molecular Biology II, Institute of Nutrition and Food Technology “José Mataix, Centre for Biomedical Research, University of Granada”, Granada, Spain; 6 Instituto de Investigación Biosanitaria ibs-Granada, Granada, Spain; 7 Physiopathology of Obesity and Nutrition Networking Biomedical Research Centre (CIBEROBN), Madrid, Spain

**Keywords:** Metabolomics, Obesity and overweight, Metabolic profiling, Weight loss, Dietary intervention

## Abstract

**Introduction:**

Obesity is a disorder characterized by a disproportionate increase in body weight in relation to height, mainly due to the accumulation of fat, and is considered a pandemic of the present century by many international health institutions. It is associated with several non-communicable chronic diseases, namely, metabolic syndrome, type 2 diabetes mellitus (T2DM), cardiovascular diseases (CVD), and cancer. Metabolomics is a useful tool to evaluate changes in metabolites due to being overweight and obesity at the body fluid and cellular levels and to ascertain metabolic changes in metabolically unhealthy overweight and obese individuals (MUHO) compared to metabolically healthy individuals (MHO).

**Objectives:**

We aimed to conduct a systematic review (SR) of human studies focused on identifying metabolomic signatures in obese individuals and obesity-related metabolic alterations, such as inflammation or oxidative stress.

**Methods:**

We reviewed the literature to identify studies investigating the metabolomics profile of human obesity and that were published up to May 7th, 2019 in SCOPUS and PubMed through an SR. The quality of reporting was evaluated using an adapted of QUADOMICS.

**Results:**

Thirty-three articles were included and classified according to four types of approaches. (i) studying the metabolic signature of obesity, (ii) studying the differential responses of obese and non-obese subjects to dietary challenges (iii) studies that used metabolomics to predict weight loss and aimed to assess the effects of weight loss interventions on the metabolomics profiles of overweight or obese human subjects (iv) articles that studied the effects of specific dietary patterns or dietary compounds on obesity-related metabolic alterations in humans.

**Conclusion:**

The present SR provides state-of-the-art information about the use of metabolomics as an approach to understanding the dynamics of metabolic processes involved in human obesity and emphasizes metabolic signatures related to obesity phenotypes.

**Electronic supplementary material:**

The online version of this article (10.1007/s11306-019-1553-y) contains supplementary material, which is available to authorized users.

## Background

Obesity is a disorder characterized by a disproportionate increase in body weight in relation to height, mainly due to the accumulation of fat. Obesity is considered a pandemic of the present century by the World Health Organization (WHO) and other international organizations (Abarca-Gómez et al. 2017; World Health Organization 2014). Obesity is associated with the development of important non-communicable chronic diseases, namely, hypertension, metabolic syndrome, type 2 diabetes mellitus (T2DM), cardiovascular diseases (CVD), obstructive sleeping apnea, osteoarthropathies and cancer (GBD 2015 Obesity Collaborators et al. 2017; Williams et al. 2015).

Worldwide, obesity has nearly tripled since 1975, and in 2016, more than 1.9 billion adults aged 18 years and older (39% of the global population) were overweight. Of these individuals, over 650 million (13% of the total population) were obese. Moreover, 41 million children under the age of five were overweight, and over 340 million children and adolescents aged 5–19 were overweight or obese (World Health Organization 2018).

Obesity is usually diagnosed by estimating the body mass index (BMI), which is calculated as the ratio of body weight (kg) and height squared (m^2^), allowing physicians to classify individuals by grade from overweight to morbid obesity (World Health Organization 2018). However, this simple and useful index does not evaluate the metabolic alterations frequently associated with obesity, which in turn are closely related to the existence of insulin resistance (IR) in peripheral tissues (Cañete et al. 2007) or immunological disorders occurring as a consequence of the establishment of a low-level inflammatory process derived from the activation of the innate immune system (Hotamisligil 2006). The latter process also leads to IR and altered glucose and lipid metabolism (Bastard et al. 2006), as well as the secretion of numerous pro-inflammatory cytokines (Tilg and Moschen 2006) and factors involved in angiogenesis and blood coagulation (Brestoff and Artis 2015; Caputo et al. 2017). Additionally, adipocyte hypertrophy induces the accumulation of reactive oxygen species due to endoplasmic reticulum dysfunction (Hotamisligil 2010) and the activation of cell inflammatory signaling cascades (Lee and Lee 2014). Many adipokines and inflammatory factors have been suggested as biomarkers of obesity (Gil-Campos et al. 2004). In fact, in obese subjects, the expression of many genes related to cell metabolism and production of adipokines is significantly altered (Aguilera et al. 2015; Gil et al. 2007; Kim and Park 2010).

Substantial controversy exists regarding whether obesity should be considered a disease (Vallgårda et al. 2017). Within the obese population, clinicians can distinguish between metabolically healthy obese (MHO) and metabolically unhealthy obese (MUHO) subjects. Increased blood pressure, hyperlipidemia, hyperglycemia, hyperuricemia and increased peripheral IR are frequently reported in MUHO subjects (Badoud et al. 2015a, b; Rupérez et al. 2018).

Metabolomics is defined as a technological tool that aims to detect and measure changes in the profiles and levels of low molecular weight metabolites (< 1500 Da) in cells, tissues, organs, systems or whole organisms in response to a genetic variation or physiological or pathological condition (Gibney et al. 2005). Therefore, metabolomics enlightens as a useful tool to evaluate changes in metabolites due to overweight and obesity at the cellular level, i.e., visceral and omental white adipose tissues (AT), brown AT, skeletal muscle, liver, among others, and body fluid level, i.e., plasma, urine, and human milk. Also, this analytical tool is of keen interest in ascertaining the metabolic fingerprint (a recognizable chemical pattern specific of an individual sample) related to metabolically unhealthy obese individuals compared to metabolically healthy individuals (Badoud et al. 2015b).

Metabolomics comprises qualitative and quantitative analyses of intracellular and intercellular metabolites, usually using two main distinct analytical approaches: (a) nontargeted metabolite profiling, intended as a comprehensive analysis without further knowledge of the features covered and which might result in the identification and characterization of a large variety of metabolites that can cluster into recognizable patterns; and (b) targeted metabolite profiling, that is focused on a reliable quantitative measurement of the variations in metabolites involved in a number of metabolic pathways (e.g., amino acids (AA) and their derivatives) based on an understanding of their biological roles in those pathways (Park et al. 2015). These methods differ in numerous aspects, such as the complexity of the sample preparation procedures, the experimental precision, the range of features (metabolites) detected, and the quantification level (relative versus absolute) (Rangel-Huerta and Gil 2016). Those characteristics prompt researchers to establish specific objectives for each approach, such as generating a hypothesis or testing a previously developed hypothesis (Putri et al. 2013).

Over the last decade, numerous reports and reviews have addressed the metabolic changes associated with obesity in both humans and animal models (Abu Bakar et al. 2015; Adams 2011; Calvani et al. 2014; Du et al. 2013; Fiehn et al. 2010; Gogna et al. 2015; He et al. 2012; Hivert et al. 2015; Kim and Park 2010; Kim et al. 2010a; Mihalik et al. 2012; Moore et al. 2013; Morris et al. 2012; Newgard 2017; Newgard et al. 2009; Oberbach et al. 2011; Pietiläinen et al. 2007; Rauschert et al. 2014, 2016; Rauschert et al. 2017; Rauschert et al. 2017; Shore and Cho 2016; Tulipani et al. 2016a; Villarreal-Pérez et al. 2014; Wahl et al. 2012; Williams et al. 2006; Xie et al. 2012; Zeng et al. 2010; Zhang et al. 2013; Zhao et al. 2016a, b). Many of them describe changes in the metabolic profile associated with obesity and diabetes, and notably features associated with IR (Abu Bakar et al. 2015; Adams 2011; Fiehn et al. 2010; Gogna et al. 2015; Mihalik et al. 2012; Newgard 2017; Newgard et al. 2009; Rauschert et al. 2016; Villarreal-Pérez et al. 2014; Zhao et al. 2016a, b), and the majority report the results of targeted analyses. Indeed, the identified metabolites can serve as biomarkers of the pathophysiological mechanisms involved in the development of obesity and, subsequently, T2DM. Elevated levels of branched-chain AAs (BCAAs) (leucine, isoleucine, and valine) and aromatic AAs (phenylalanine, tyrosine, tryptophan and methionine), as well as some of their tissue metabolites, have been detected in both subjects with obesity and diabetes, whereas glutamine and glycine levels are decreased (Adams 2011; Mihalik et al. 2012; Morris et al. 2012; Newgard 2017; Newgard et al. 2009; Rauschert et al. 2017), although the results have not always been consistent (Fiehn et al. 2010; Kim et al. 2010b; Oberbach et al. 2011; Wahl et al. 2012). The levels of other non-protein nitrogen compounds, such as nucleotides, nucleosides, and their metabolites, namely, uridine and uric acid, vary considerably, depending on the degree of IR in obese subjects (Fiehn et al. 2010; Park et al. 2015; Wahl et al. 2012). Regarding lipid metabolites, the levels of some fatty acids (FAs), e.g., palmitic, palmitoleic, stearic, and oleic acids, and stearoyl carnitine are elevated in obese subjects (Park et al. 2015). Likewise, the levels of some lysophospholipids of both choline and ethanolamine seem to be altered, although the direction of changes is not consistent and depend on each study (Fiehn et al. 2010; Gogna et al. 2015; Kim et al. 2010b; Moore et al. 2013; Pietiläinen et al. 2007; Wahl et al. 2012). Concerning carbohydrates, the concentrations of glucose, fructose, mannose, xylose, gluconic acid, glucuronic acid, glycerol and lactate in plasma are usually increased, whereas the concentrations of glycerol-3-phosphate and other metabolites are decreased in obese men (Fiehn et al. 2010; Gogna et al. 2015; Moore et al. 2013; Park et al. 2015). A summary of the changes in the major metabolites in subjects with obesity and diabetes obtained using a targeted metabolomics approach has been previously reported (Putri et al. 2013).

In this context, we aimed to perform a systematic review (SR) of human studies focused on identifying metabolomic signatures in obese individuals and obesity-related metabolic alterations, such as inflammation or oxidative stress; we considered the targeted and nontargeted approaches as different and separate strategies within the metabolomics analyses. Furthermore, we included studies evaluating the metabolic signature and its modulation by dietary interventions, such as dietary challenges or weight loss programs, in humans.

## Methods

The present SR was designed to review the state-of-the-art research related to the use of metabolomics as an approach to understanding the dynamics of metabolic processes involved in human obesity.

**Fig. 1:**
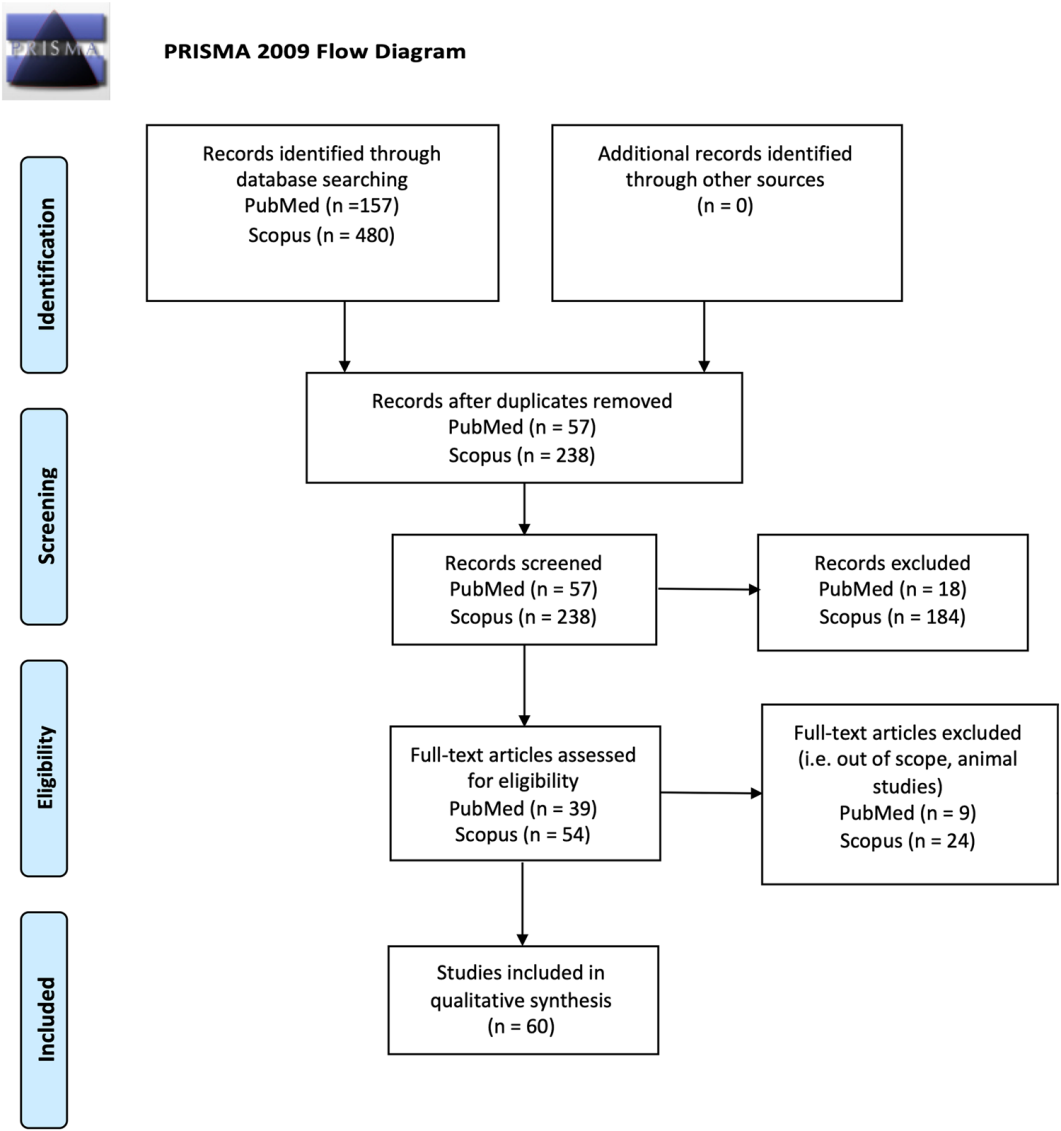
PRISMA 2009 flow diagram

This review was conducted following the PRISMA-P (Preferred Reporting Items for SR and Meta-Analysis Protocols) statement (Moher et al. 2015) (see Fig. 1).

### Inclusion and exclusion criteria

Studies employing cross-sectional, prospective, parallel, and crossover designs were considered. The sample size was not restricted. Articles, or at least the abstract, must have been written in English or Spanish. Conference abstracts, reviews, meta-analyses, case reports, ecological studies, and letters to the editor were excluded.

### Search strategy and eligibility criteria

Studies should have focused on the metabolic profile of obese humans or their regulation by weight loss interventions, dietary products, or dietary challenges to be considered for inclusion in the SR. Studies of overweight or obese subjects in which the outcomes were strictly related to the study of obesity were included. All the studies utilizing a metabolomics approach, including but not limited to nuclear magnetic resonance (NMR) or MS (coupled to different types of chromatography) of urine or plasma samples, were included. Studies published up to May 7th, 2019, were included.

### Literature search

We performed an SR of studies published in English or Spanish of the populations included in the following electronic databases without an age limit: MEDLINE (via PubMed) and SCOPUS. Figure 1 shows the main steps of the literature search. Studies were identified in the databases by applying a publication date of May 7th, 2019, human subjects, and the following search MeSH terms (exclusive of PubMed): (“Metabolome”[Majr] OR “Metabolomics”[Majr]) AND “Obesity”[Majr] AND “humans”[MeSH Terms].

MeSH terms are restricted to medical databases such as PubMed. Therefore, as SCOPUS has more extensive coverage than PUBMED, the search was done using metabolome, metabolomics, and other additional terms. The search in SCOPUS was filtered by articles using the following equation: (“obesity” OR “overweight”) AND (“metabolic profiling” OR “metabolic fingerprint” or metabolomics).

### Study selection and data extraction

First, both the titles and abstracts of publications yielded by the search were reviewed independently by two reviewers, ODHR and BPV and studies that did not meet the established language, subject matter, participant, design and outcome criteria were excluded (see Fig. 1).

ODHR and BPV input the data into the database; one additional reviewer (AG) resolved any discrepancies. After selecting the final list of articles to be included, the authors elaborated a classification according to the objectives and approach of the studies to facilitate the interpretation of the results.

### Quality assessment

The quality of the selected articles included in the present SR was evaluated using the QUADOMICS tool, which has been employed in other metabolomics reviews (Lumbreras et al. 2008; Zhang et al. 2017; Zhao et al. 2016a, b). This tool was developed to evaluate quality issues specific to omics research and has been used to assess the quality of diagnostic studies in a highly dynamic field that faces the challenge of sieving many recently published results (Lumbreras et al. 2008). Because of the wide range of types of studies, we selected specific items that covered the major domains. The items and the evaluation criteria are included as Supplementary Material (Supplemental Table 1).

**Table 1:** Studies examining the metabolic profiles associated with human obesity using targeted and untargeted approaches

Author (year)	Population (tissue)	Characteristics	Platform	Statistical analysis	Outcome
Untargeted approaches					
Fattuoni et al. (2018)	Adult women (placenta)	20 normal-weight and 18 obese women	GC–MS	PLS-DA	Metabolic signature of obesity
Ruebel et al. (2019)	Adult women (follicular fluid)	8 overweight/obese and 9 normal-weight	GC-q-ToF and LC–MS/MS	PLS-DA	Metabolic signature
Houttu et al. (2018)	Adult (serum)	52 overweight and 47 obese pregnant women	NMR	Mann–Whitney U test, PCA, Spearman correlation	Metabolic signature of obesity in pregnancy
Sorrow et al. (2019)	Children (umbilical cord)	25 obese and 25 matched non-obese	LC–MS/MS Metabolon	Logistic regression	Metabolic signature of obesity: prediction
Butte et al. (2015)	Children (plasma)	353 non-obese children (190 women) and 450 obese children (208 women)	GC–MS and UHPLC-MS	Random forest and PCA	Signature of obesity
Kim et al. (2010b)	Adults (serum and plasma)	30 non-obese males and 30 overweight/obese males	GC–MS and UPLCQ-TOF-MS	PLS-DA	Characterization of obesity
Xie et al. (2014)	Adults (serum)	105 non-obese subjects (67 women) and 106 obese subjects (67 women)	GC-TOF-MS and UPLCQ-TOF-MS	PCA, OPLS-DA	Signature of obesity (BCAAs)
Hanzu et al. (2014)	Adults (visceral and subcutaneous adipose tissue)	6 non-obese subjects (four women) and 8 morbidly obese subjects (6 women)	GC–MS	PCA and ANOVA	Characterization of obesity
Zhao et al. (2016a, b)	Adults (plasma)	77 normoglycaemic non-obese subjects (48 women) and 354 normoglycaemic overweight-obese subjects (231 women)	LC–MS	sPLS-DA	Signature of obesity
Foerster et al. (2015)	Adults (serum)	226 patients (120 women)	Two-dimensional GC (coupled to TOF-MS) and lipids (ultra-performance LC–MS)	Treelet transform and PCA	Association with anthropometry
Bagheri et al. (2019)	Adults (plasma)	200 obese patients and 100 healthy controls	LC–MS/MS	Multivariable linear regression	Obesity metabolic signature
Cirulli et al. (2019)	Adults (serum)	1743 adults (twins) and 427 for the validation	UPLC-MS/MS	Linear regression	Obesity metabolic signature
Yu et al. (2018)	Adults (serum and urine)	36 overweight/obese and 35 normal-weight men	UPLC-Q-TOF-MS	PCA and PLS-DA	Explore the primary endogenous metabolic alterations in the early phase of obesity
Marco-Ramell et al. (2018)	Adults	64 individuals (19 men and 45 women)	LC–MS/MS	OPLS-DA	Metabolic signature of obesity
Piening et al. (2018)	Adults (plasma)	23 non-obese subjects	LC–MS	Univariate analysis	Characterization of weight gain and loss
Targeted approaches					
Wahl et al. (2012)	Children (serum)	40 non-obese subjects (15 women) and 80 obese subjects (38 women)	LC–MS/MS	PLS and logistic regression	Characterization of obesity
Gawlik et al. (2016)	Children (24-h urine)	87 obese children (44 women)	GC–MS	K-Means clustering (metaboanalyst) and ANOVA	Steroid signature in obese children
Newgard et al. (2009)	Adults (serum and urine)	67 non-obese (38 women), 74 obese subjects (52 women)	GC–MS, MS/MS	PCA and Wilcoxon rank-sum testing	Characterization of obesity
Baker et al. (2015)	Adults (skeletal muscle and plasma)	6 non-obese and 6 obese males	LC–MS/MS	ANOVA	Signature of obesity (effects of obesity and 5 days of HFD in the 4 h postprandial condition)
Kraus et al. (2016)	Adults (plasma)	111 non-obese and 628 obese subjects (431 women)	MS-Q-ToF	PCA	Metabolic signature and BMI
Feldman et al. (2019)	Adults (serum)	69 non-obese and 50 healthy obese	LC–MS/MS (Biocrates p180 kit)	*T* test using FDR adjustment (Benjamini-Hochberg)	Characterization of obesity
Maltais-Payette et al. (2018)	Adults (plasma)	59 non-obese middle age-women	LC–MS/MS (Biocrates p180 kit)	ANOVA, Pearson correlation	Investigate the role of glutamate as a predictor of visceral obesity and metabolic wellness
Carayol et al. (2017)	Adults (plasma)	392 subjects from the EPIC-Oxford cohort and 327 control subjects	LC–MS/MS (Biocrates p180 kit)	PCA and linear regression	Metabolic profiling and BMI
Bagheri et al. (2018)	Adults (plasma)	107 metabolic healthy obese, 100 metabolic unhealthy obese and 78 non-obese	Targeted LC–MS	PCA	Characterization of MHO and MUHO
Wang et al. (2018)	Adults (serum)	302 overweight/obese and 298 non-obese	Targeted LC–MS	Correlation, multiple linear and logistic regression analyses	Metabolic signature of obesity
Tulipani et al. (2016a)	Adults (serum)	31 non-obese subjects (23 women) and 33 morbidly obese subjects (22 women) (both classified based on the risk of developing T2D)	LC- and FIA-ESI–MS/MS	ANOVA, HSD Tukey contrasts, regression, DLDA, LDA, QDA, PLS-DA, and SCDA	Signature of obesity and risk of T2D
Ho et al. (2016)	Adults (plasma)	1787 non-obese and 596 obese subjects (1264 women)	LC/MS	PROC GLIMMIX	Associations between metabolites obesity (BMI and IR)
Haufe et al. (2016)	Adults (plasma)	111 overweight to obese subjects	GC–MS and LC–MS/MS	Simple and partial correlations	Metabolic signature and BMI/IR
Stroeve et al. (2016)	Adults (plasma)	667 overweight, obese, or MO individuals (431 women)	NMR (targeted) and LC–MS (lipid targeted)	PLS-DA	Changes in metabolomic profile and predictive tool
Cho et al. (2017)	Adolescents (urine)	91 non-obese subjects (44 women) and 93 obese subjects (40 women)	LC-Q-TOF (untargeted), LC–MS/MS, and FIA-MS/MS (targeted)	PCA, Wilcoxon signed rank test, simple correlation, and linear regression	Signature of obesity

*AA* amino acids, *BCAA* branched-chain amino acids, *BMI* body mass index, *DLDA* diagonal discriminant analysis, *FAs* fatty acids, *FDR* false discovery rate, *FFAs* free fatty acids, *FIA* flow injection analysis, *GC* gas chromatography, *HFD* high-fat diet, *IR* insulin resistance, *LDA* linear discriminant analysis, *MO* morbidly obese, *MS* mass spectrometry, *NMR* nuclear magnetic resonance, *OPLS*-*DA* orthogonal partial least square discriminant analysis, *PC* phospholipids, *PLS*-*DA* partial least squares projection to latent structures-discriminant analysis, *QDA* quadratic discriminant analysis, *Q*-*TOF* quadrupole-time of flight, *SCDA* nearest shrunken centroid classification, *UPLC* ultra-high performance liquid chromatography, *T2D* type 2 diabetes

## Result

### Selection of metabolomics studies investigating obesity

The process for the selection of studies after the literature search is described in Fig. 1. Finally, we reviewed 60 studies that met established inclusion criteria and were evaluated by quality according to the QUADOMICS evaluation (see Supplemental Table 1). According to the type of approach reported on the studies, we have divided the results into four blocks. The first block includes studies designed to determine the metabolic signature of obesity; 15 of which used an untargeted approach (Fattuoni et al. 2018; Ruebel et al. 2019; Houttu et al. 2018; Sorrow et al. 2019; Butte et al. 2015; Kim et al. 2010b; Xie et al. 2014; Hanzu et al. 2014; Zhao et al. 2016a, b; Foerster et al. 2015; Bagheri, et al. 2019, Cirulli et al. 2019, Yu et al. 2018, Marco-Ramell et al. 2018, Piening et al. 2018), 14 used targeted metabolite profiling (Wahl et al. 2012; Gawlik et al. 2016; Newgard et al. 2009; Baker et al. 2015; Kraus et al. 2016; Feldman et al. 2019; Maltais-Payette et al. 2018; Carayol et al. 2017; Bagheri et al. 2018; Wang et al. 2018; Ho et al. 2016; Haufe et al. 2016; Stroeve et al. 2016; Tulipani et al. 2016a, b), and one designed the metabolomics study using a combination of both approaches (Cho et al. 2017) (Table 1). The second block includes five studies focused on studying the differential responses of obese and non-obese subjects to dietary challenges (Table 2) (Badoud et al. 2015b; Baker et al. 2015; Geidenstam et al. 2014; Bak et al. 2018). The third block comprises three studies that used metabolomics to predict weight loss (Geidenstam et al. 2017a, b; Stroeve et al. 2016) and 11 randomized clinical trials (RCTs) aimed to assess the effects of weight loss interventions (both hypocaloric diet programs and exercise interventions) on the metabolomic profiles of overweight or obese human subjects (Table 2) (Almanza-Aguilera et al. 2018; Duft et al. 2017; Kang et al. 2018; Leal-Witt et al. 2018; Meucci et al. 2017; Mills et al. 2019; Munukka et al. 2018; Palau-Rodriguez et al. 2019; Perez-Cornago et al. 2014; Zheng et al. 2016a, b). Additionally, the fourth block includes 11 articles that studied the effects of specific dietary patterns or dietary compounds on obesity-related metabolic alterations in humans, such as inflammation or oxidative stress (Table 3) (Baldrick et al. 2018; Gu et al. 2013; Hernández-Alonso et al. 2019; Hibberd et al. 2019; Kim et al. 2013; Kim et al. 2017; Mayengbam et al. 2019; Nieman et al. 2012a, b; Romo-Hualde et al. 2018; Xu et al. 2018).

**Table 2:** Metabolomics studies focused on studying the differential response of obese and non-obese subjects to dietary challenges and weight loss

Author	Population	Biospecimen (platform)	Intervention	Duration	Outcome
Differences in response to dietary intake challenges					
Badoud et al. (2015a, b)	10 lean healthy adults ten MHO adults ten MUO adults	Plasma (CE–MS, GC–MS)	High-calorie meal (including two sausage egg english muffins, one apple turnover and 370 ml of concentrated orange juice, 1330 kcal)	Acute intervention (120 min)	AAs and FAs profile
Geidenstam et al. (2014)	14 obese adults with impaired glucose tolerance	Serum (targeted GC–MS)	0, 30 and 120 min during a standard 75 g OGTT	Acute intervention (120 min)	Differences in response to an OGTT between morbidly obese and lean individuals
Geidenstam et al. (2016)	14 obese adults with impaired glucose tolerance	Serum (GC-TOF-MS)	0, 30 and 120 min during a standard 75 g OGTT after weight loss and after weight maintenance.	Acute intervention (120 min)	Differences in response to an OGTT between morbidly obese and lean individuals
Baker et al. (2015)	6 male non-obese adults six male obese adults	Skeletal muscle, plasma (targeted LC–MS/MS)	HFD in the 4 h postprandial condition	5 days	Differences in response to HFD in AA, short-chain acylcarnitines
Bak et al. (2018)	9 lean men nine obese men	Skeletal muscle (UHPLC/MS/MS)	Fasting	12 and 72 h of fasting	To explore and compare substrate metabolism in skeletal muscle
Prediction of weight loss					
Geidenstam et al. (2017a)	12 weight loss and weight maintenance cohort (WLWM) replication cohort of 83 obese adults	Plasma, serum (GC–MS)	(1) WLWM: Low-calorie diet (1200 kcal/day) for three monts; followed by a 6 months weight maintenance program (2) replication cohort: behavioral therapy and whenever possible proceeded by a prolonged period with a low-calorie diet	9 months	Identify predictors of weight loss: Study and validate changes in metabolite levels associated with moderate weight loss
Geidenstam et al. (2017b)	91 obese adults n = 58 > 10% weight loss n = 33 < 10% weight loss	Serum (GC–MS, LC–MS/MS)	(1) Weight loss program (classified according < or > 10% weight loss)	1 year	Identify predictors of weight loss: study and validate changes in metabolite levels associated with moderate weight loss
Stroeve et al. (2016)	667 overweight, obese, MO adults (431 women)	Plasma (targeted NMR, LC–MS)	(1) Low-calorie diet (800 kcal)	8 weeks	Changes in metabolomics profile and predictive tool
Changes related to weight loss intervention					
Leal-Witt et al. (2018)	34 obese prepubertal children (15 women)	Urine (NMR)	Lifestyle intervention program (following the Mediterranean diet and WHO recommendations + physical activity increment)	6 months	To identify metabolic signatures associated with lifestyle intervention
Kang et al. (2018)	97 overwight adults (70 women)	Plasma (UPLC-LTQ-Orbitrap MS)	(1) Low-calorie diet (2) control (weight maintenance diet)	12 weeks	Changes in metabolomics profile
Palau-Rodriguez et al. (2019)	27 MHO women	Plasma (UPLC-ESI-MS/MS)	(1) Hypocaloric Mediterranean diet and physical activity*	12 months	To identify metabolic fingerprints and their associated pathways, according to level of weight loss: < 10% (LWL) and > 10% (HWL)
Mills et al. (2019)	1158 obese pregnant women	Serum (NMR)	(1) Diet and physical activity intervention from UPBEAT (2) control (standard antenatal care)	During pregnancy and up to 6 months	Changes in metabolomics profile
Perez-Cornago et al. (2014)	22 overweight and obese adults	Serum (GC–MS)	(1) Energy-restricted diet (−15% of daily energy requirements)	8 weeks	Changes in FA and AA profiles
Almanza-Aguilera et al. (2018)	57 MHO adult women	Plasma (H NMR)	(1) Lifestyle weight loss treatment group (hypocaloric Mediterranean diet and regular physical activity) (2) control group (general recommendations of a healthy diet and physical activity)	3 months (intervention) and 12 months (follow-up)	Changes in metabolomics profile
Zheng et al. (2016a, b)	38 overweight or obese adult women	Urine, plasma and faeces (NMR)	(1) Energy-restricted Intervention (500 kcal energy deficit/day) + low-dairy (2) energy-restricted intervention (500 kcal energy deficit/day) + high-dairy intake	24 weeks	Changes in metabolomics profile
Zheng et al. (2016b)	POUNDS LOST study (526 adults)	Plasma (targeted EI–MS/MS)	Weight loss interventions	6 months and 2 years	Long-term dietary intervention for weight loss affects circulating AA
Zheng et al. (2016b)	DIRECT study (211 overweight or obese adults)	Plasma (targeted EI-MS/MS)	(1) Low-fat, restricted-calorie diet (2) Mediterranean, restricted-calorie diet (3) low-carbohydrate, nonrestricted-calorie diet	6 months and 2 years	Long-term dietary intervention for weight loss affects circulating AA
Changes related to weight loss through physical activity					
Munukka et al. (2018)	17 overweight women	Plasma and faeces (NMR)	Endurance training (3 training sessions/week)	6 weeks	Changes in the gut metagenome and systemic metabolites
Meucci et al. (2017)	22 overweight preadolescents	Urine (GC-TOF-MS)	(1) 8-week exercise program (2) 4-week exercise program (3) control	8 weeks	8 weeks of activity as cause the greatest shift in the metabolites
Duft et al. (2017)	22 obese adults’ subjects	Serum (H NMR)	(1) Control group (n = 11) (2) combined training (n = 11)	24 weeks (3 times/week)	Changes in metabolism after 24 weeks of combined training

*AA* amino acids, *AEE* activity energy expenditure, *AUC* area under the curve, *CE* capillary electrophoresis, *EI* electronic ionization, *FA* fatty acids, *H NMR* proton nuclear magnetic resonance, *HWL* high weight loss group, *GC* gas chromatography, *LC* liquid chromatography, *LDL* low-density cholesterol, *LWL* low weight loss, *MetS* metabolic syndrome, *MHO* metabolic healthy obese, *MO* morbidly obesity, *MS* mass spectrometry, *MUO* metabolically unhealthy obese, *NMR* nuclear magnetic resonance, *OGTT* oral glucose tolerance test, *Q*-*TOF* quadrupole-time of fligh, *UPBEAT* better eating and activity trial, *UPLC* ultra-performance liquid chromatography

*Based on a reduction of about 600 kcal in the energy intake with a calorie distribution as follows: 35–40% fats (8–10% saturated fatty acids), 40–45% carbohydrates and 20% protein + exercise (walking on average for 150 min every week)

**Table 3:** Metabolomics studies from dietary and supplement interventions in obesity and risk related factors

Author	Tissue (biospecimen)	Characteristics	Intervention	Duration	Metabolomics platform	Outcome
Nieman et al. (2012a)	Plasma	62 overweight adult women	(1) 25 g/day milled chia (2) 25 g/day whole chia (3) 25 g/day placebo	10 weeks	GC–MS	Targeted metabolites of inflammation and disease risk factors
Nieman et al. (2012b)	Serum	98 overweight and obese adult women	(1) 1 g/day red pepper spice (2) 2.8 g/day turmeric (3) placebo	4 weeks	GC–MS	Changes in inflammation and oxidative stress metabolites
Gu et al. (2013)	Serum	(1) 45 healthy obese adults (2) 30 healthy control adults	(1) Very low-carbohydrate diet	8 weeks (0, 4- and 8-weeks’ time points)	UPLC-Q-TOF-MS	Changes in FA, AA, carbohydrates profile
Kim et al. (2013)	Serum	34 overweight/obese adults (19 women)	(1) Dietary intervention plus 4.5 g of black soybean peptides (2) placebo	12 weeks	UPLC-Q-TOF-MS	To identify key metabolites related to weight reduction
Kim et al. (2017)	Plasma and urine	72 sedentary overweight/obese adults	(1) 30 g/day KBR* (2) 30 g/day NAB (3) Placebo (all with an exercise challenge)	4 weeks	H NMR	To identify metabolites that predict responses to an intervention against oxidative stress and inflammation
Baldrick et al. (2018)	Serum and urine	80 overweight/obese adults (41 women)	(1) 400 mg capsule (100 mg seaweed (poly)phenol) (2) placebo (400 mg maltodextrin)	8 weeks	UHPLC-HR-MS	Effects on oxidative damage to DNA, plasma antioxidant capacity, inflammatory responses and chronic low- level inflammation
Romo-Hualde et al. (2018)	Urine	70 overweight/obese women	(1) EPA (1.3 g/day) (2) α-LA (0.3 g/day) (3) EPA + α-LA (1.3 g/day + 0.3 g/day) (4) control	10 weeks	HPLC-TOF-MS	Changes in metabolomics profile. Role of the antioxidant properties
Xu et al. (2018)	Plasma	19 obese adults with MetS (five women)	(1) Low-fat milk (2) rice milk	Postprandial	HPLC-MS/MS	Differences in metabolomics profile and to identify metabolic pathways
Hernández-Alonso et al. (2019)	Plasma	122 overweight/obese adults (82 women)	(1) LGI diet (2) HGI diet (3) LF diet (all groups: 500-kcal energy-restricted)	6 months	H NMR, GC-Q-TOF, LC-Q-TOF	Improvement of metabolites profile (AA and lipids) by LGI diet versus HGI diet and LF diet
Hibberd et al. (2019)	Plasma and faeces	134 healthy overweight or obese (103 women)	(1) 12 g/day LU; (2) 1010 cfu/day *Bifidobacterium animalis* subsp. *lactis* 420™ (B420) in 12 g microcrystalline cellulose; (3) 10^10^ cfu/d of B420 in 12 g/d LU (4) Placebo, 12 g/day microcrystalline cellulose	6 months	NMR, UPLC-MS/MS	Association of changes in the gut microbiota, faecal metabolites and bile acid metabolism with the observed clinical benefits
Mayengbam et al. (2019)	Serum and faeces	53 overweight/obese adults (43 women)	(1) 15 g/day pea fibre in wafer form (2) control with isocaloric number of wafers	12 weeks	H NMR, FIA-MS/MS	Changes in microbiome, faecal BA and SCFA profile (microbiome–host–metabolic axes in obesity)

*AA* amino acids, *BA* bile acids, *BCAA* branched-chain amino acids, *EPA* eicosapentaenoic acid, *FA* fatty acids, *FIA* flow injection analysis, *GC* gas chromatography, *HGI* high glycemic index, *H NMR* proton nuclear magnetic resonance, *HPLC* high performance liquid chromatography, *HR* high-resolution, *KBR* Korean black raspberry, *LA* lipoic acid, *LC* liquid chromatography, *LF* low-fat, *LGI* low-glycaemic index, *LU* Litesse^®^ Ultra™ polydextrose, *MetS* metabolic syndrome, *MS* mass spectrometry, *NAB* Northern American black raspberry, *NMR* nuclear magnetic resonance, *Q*-*TOF* quadrupole time of flight, *SCFA* short-chain fatty acids, *TCA* tricarboxylic acid, *UPLC* ultra-high performance liquid chromatography

*Daily dose of KBR represented 0.9 g of total phenol, including 17.5 mg of myricetin, 9.6 mg of genistein, 7.2 mg of quercetin, 1.2 mg of daidzein, and 1.2 mg of eriodictyol, as well as 126 kcal (65.5% as carbohydrate, 10.1% as protein, and 5.4% as fat). The daily dose of AB represented 1.3 g of total phenol, including 25.2 mg of myricetin, 16.6 mg of genistein, 7.4 mg of kaempferol, 3.9 mg of quercetin, 1.8 mg of eriodictyol, and 0.6 mg of daidzein, as well as 111 kcal (67.8% as carbohydrate, 7.5% as protein, and 7.6% as fat)

### Metabolomic profiling of obesity

#### Untargeted analysis

Three studies focused on profiling the obesity during pregnancy (Table 1). The analysis of placenta samples from obese women reveals a potentially altered metabolism as reflected by the dysregulation of several pathways. Metabolites associated with the antioxidant defense system, nucleotide production, as well as lipid metabolism and energy production were modified. A specific serum fatty acid profile characterized by low levels of LC-PUFA derivatives, arachidonic acid, and DHA, and high levels of palmitic acid were observed (Fattuoni et al. 2018).

Furthermore, the study of the follicular fluid from overweight and obese women presented similar oxidative stress and lipid metabolism alterations. Elevated concentrations of uric acid and several unknown lipids, as well as a decrease of 2-ketoglucose dimethyl acetal, aminomalonate, two unknown primary metabolites, and two unknown complex lipids in the overweight and obese woman (Ruebel et al. 2019). Moreover, using NMR, serum metabolic profiling revealed a different lipid profile in pregnant women when compared with overweight. Specifically, the obese pregnant women showed significantly higher very low-density lipoprotein (VLDL) and lower high-density lipoprotein particles (HDL); lower proportions of ω-6 fatty acid, 18:2 linoleic acid and PUFAs of total FAs, as well as the estimated degree of unsaturation of FAs (Houttu et al. 2018).

Two studies performed untargeted metabolomics analysis in children (Sorrow et al. 2019; Butte et al. 2015), (Table 1). First, the study of the umbilical cord from children developing obesity by age 3–5 years old provided valuable information (Sorrow et al. 2019). Those children with elevated concentrations of medium and very long-chain FAs (LCFAs), such as stearate, oleate or palmitate at birth, developed obesity later in life. Moreover, the authors reported an association between obesity and several acetaminophen metabolites at birth, including 3-(N-acetyl-l-cysteine-S-yl) acetaminophen, 2-hydroxyacetaminophen sulfate, 2-methoxyacetaminophen glucuronide, and p-acetamidophenyl glucuronide.

Butte et al. (2015) reported that the relative plasma concentrations of BCAAs (leucine, isoleucine, and valine), their catabolites (2-methylbutyrylcarnitine, 3-methyl-2-oxobutyrate, and isovalerylcarnitine), propionylcarnitine (C3) and butyrylcarnitine (C4) were significantly increased in obese children compared with non-obese children (Butte et al. 2015). The authors (Butte et al. 2015) also observed increased levels of both polar and non-polar AAs (glutamate, lysine, tyrosine and phenylalanine, and alanine, respectively), polyamines, several gamma-glutamyl dipeptides and polypeptides in obese children. In contrast, asparagine, aspartate, glycine, serine, and histidine levels were decreased. Notably, significantly higher levels of the ketone bodies containing α-hydroxybutyrate and α-ketobutyrate were observed in the obese children, but lower concentrations of lysophospholipids (glycerophosphocholines and glycerophosphoethanolamines) and decarboxylated FAs (dodecanedioate, tetradecanedioate, and 2-hydroxydecanoate) were observed compared with non-obese children. Significantly lower β-hydroxybutyrate levels were also observed in obese children than in non-obese children. Furthermore, markedly higher levels of steroids, such as dehydroepiandrosterone sulfate (DHEA-S), were detected in obese children. Additionally, higher mannose and pyruvate levels and lower glycerate and citrate levels were measured in obese children than in non-obese children. Higher levels of purine and pyrimidine metabolites were observed in obese children. Remarkably, tyrosine was considered the highest-ranked metabolite based on its contribution to the obesity classification (Butte et al. 2015).

Regarding adults, 11 untargeted studies were included in the present SR (Table 1). In 2010, Kim et al. (2010b) reported higher levels of stearic acid and lower levels of oleic acid among the serum phospholipids of overweight/obese men. Furthermore, they also identified higher concentrations of lysophosphatidylcholine (lysoPC) a C14:0 and lysoPC a C18:0 and lower levels of lysoPC a C18:1 than in lean subjects, and confirmed eight known metabolites for overweight/obesity men (two BCAAs (valine and leucine); two essential AAs (phenylalanine and tryptophan)), as well as critical compounds of FA synthesis and oxidation (carnitine, propionyl-, butyryl-, and hexanoyl-carnitine) (Kim et al. 2010b). Using similar platforms, Xie et al. (2014) also reported higher serum BCAA levels in obese men than in lean men; moreover, BCAA levels correlated with IR and were higher in obese men, but not in obese women. Interestingly, they also reported high levels of propionyl-L-carnitine (C3) in obese men (Xie et al. 2014). More recently, Yu et al. (2018) found that obese men presented higher concentrations in serum of phenylalanine, Phe–Phe, and tryptophan, and lower levels of p-cresol and p-cresol sulfate. Interestingly, the levels of phenylacetamide, l-glutamine, phenylacetylglutamine, indoxyl sulfate, p-cresol, and p-cresol sulfate were higher in the urine of obese men.

The profiles between obese males and obese females differed, namely, higher concentrations of creatine, palmitic acid, myristic acid, n-dodecanoic acid, cis-11,14-eicosadienoic acid and linoleic acid and lower concentrations of several lysoPCs (e.g., lysoPC a C18:2, lysoPC a C20:4, and lysoPC a C20:5), uric acid and 12α-hydroxy-3-oxochola-dienic acid in females than in males (Xie et al. 2014).

Regarding the study of AT metabolites (Table 1), Hanzu et al. (2014) observed higher levels of glutamine and alanine in the visceral AT of obese subjects, as well as decreased uptake of essential AAs (methionine, threonine, and lysine), BCAAs and serine. Also, depletion of α-ketoisocaproic (α-KIC) acid was observed in the subcutaneous AT (Hanzu et al. 2014).

Zhao et al. (2016a, b) reported the associations between several measures of obesity and diverse groups of plasma metabolites. A positive correlation between the levels of oleoylethanolamide (fatty amide) and the tryptophan derivative kynurenine, and negative correlations between mannosyl-diinositol-phosphorylceramide (sphingolipid) levels with both BMI and waist circumference (WC). Moreover, auxin A (prenol lipid) and 12-ketoporrigenin levels were also positively correlated with BMI, whereas glutamate, Gly-Val-Arg-Gly peptide, pristanic acid (prenol lipid), and spirolide E (prenol lipid) levels were associated with WC (Zhao et al. 2016a, b).

Foerster et al. (2015) studied the relationship between obesity and the serum metabolome identifying two groups of compounds (compared with principal components) related to obesity. One included BCAAs and the other AA derivatives. These components were directly associated with weight, WC, BMI, body fat mass, and waist-to-height ratio. While another study (Piening et al. 2018) associated a metabolic signature comprised of 133 metabolites, mainly acylcarnitines (AC), FA, and lysophospholipids with BMI. The evidence from a large twin cohort study (Cirulli et al. 2019), provided a 49-metabolites signature (with similar compounds to those reported by Piening et al. (2018)) associated with BMI.

A recent report from Bagheri et al. (2019) identified a metabolic pattern (including 19 metabolites) associated with obesity. From such a pattern, compounds like alanine, glutamic acid, proline, tyrosine, diacyl-phosphatidylcholines, and LPCa C16:1 and BCAAs were higher in the obese participants, while asparagine, serine, acyl-alkyl-phosphatidylcholines, and other lysoPC were higher in non-obese subjects.

Marco-Ramell et al. (2018) studied the signature of obesity that overlaps with IR, and their design let them identify those compounds specific to the former. The authors reported that the presence of arachidonic, hydroxyeicosatetraenoic (HETE), palmitoleic, triHETE and glycocholic acids, HETE lactone, leukotriene B4 and two glutamyl-peptides conform a unique signature of obesity rather than of IR.

#### Targeted analysis

In children, one study (Table 1) described significantly decreased serum concentrations of the acyl-alkyl phosphatidylcholines (PC aa C34:1, PC aa C34:2, PC aa C34:3, PC aa C36:2, PC aa C36:3 and PC ae C38:2) and lysoPCs (lysoPC a C18:1, lysoPC a C18:2, and lysoPC a C20:4) in an obese group compared with lean subjects (Wahl et al. 2012). Moreover, significantly lower levels of the AAs glutamine, methionine, and proline were detected. In contrast, significantly higher concentrations of two AC (C12:1 and C16:1) were observed in obese children than in normal-weight children (Wahl et al. 2012).

Gawlik et al. (2016) aimed to identify steroid signatures in 24-h urine samples from obese children (Table 1). However, the authors did not identify any steroid profiles related to obesity status (Gawlik et al. 2016).

Regarding the adult population (Table 1), Newgard et al. (2009) observed higher levels of ethyl malonate and lower levels of isobutyryl glycine, isovaleryl glycine, and α-ketoglutarate in urine samples from obese subjects than in lean subjects (Newgard et al. 2009). Moreover, when focusing on BCAAs (Table 1), the authors reported a signature in obese subjects marked by dramatically increased concentrations of phenylalanine, alanine, valine, leucine/isoleucine, tyrosine, glutamate/glutamine, aspartate/asparagine, and arginine, whereas glycine levels appeared to be decreased. Similarly, Kraus et al. (2016) observed an inverse correlation between plasma glycine levels and BMI. While plasma phenylalanine levels have been found to be positively correlated with BMI (Ho et al. 2016) (Table 1), lower levels of other AAs, such as glycine, histidine, methionine and citrulline, have been reported in the skeletal muscle of obese subjects (Baker et al. 2015) (Table 1). Moreover, higher plasma concentrations of short-chain AC species (SCAC, C3, C4/4i, C5, and C5:1) were detected in obese subjects (Baker et al. 2015).

Additionally, Ho et al. (2016) reported positive correlations between BMI and multiple metabolites in the citric acid cycle (isocitrate, α-ketoglutarate, and aconitate), the tryptophan pathway (kynurenine and kynurenic acid), the urea cycle (citrulline and ornithine), nucleic acid metabolism (xanthosine and uric acid), and creatine-related metabolites (carnitine, choline and glycerophosphocholine). Haufe et al. (2016) also identified a positive correlation between tyrosine levels and the intrahepatic fat content and correlations between tryptophan and valine levels with hepatic IR.

In plasma samples from women, significantly lower BCAA concentrations were measured with NMR in subjects with moderate-high (27 ≤ BMI < 40 kg/m^2^) obesity than in morbidly obese (MO) subjects. Alanine, proline, and tyrosine concentrations were lower in women with moderate-high obesity than in MO women. Significant differences in the plasma levels of phosphatidylcholine species, such as lysoPC a C18:2, PC ae (34:3), PC ae (38:7), PC ae (40:6), PC ae (38:3), PC ae (40:4), and PC ae (40:8), were quantified between male and female subjects with MO and moderate-high obesity (Stroeve et al. 2016).

Cho et al. (2017) aimed to distinguish the urinary metabolomic characteristics between young obese and normal-weight subjects (Table 1). Docosaenoic acid, 12-oxo-20-carboxy-leukotriene B4, and 4α-hydroxymethyl-5α-cholesta-8-en-3β-ol levels were strongly correlated with the BMI and cholesterol levels. However, in their targeted study using the commercial solution Absolute IDQ p180, higher levels of several AC (e.g., C3, C3-DC-M/C5-OH, C3-OH, C6:1, and C8); AAs (aspartate and histidine); biogenic amines, such as asymmetric dimethylarginine (ADMA), 3,4-dihydroxyphenylalanine, putrescine and total dimethylarginine; glycerophospholipids such as lysoPC a C18:0, PC aa C38:0, PC aa C38:6, PC aa C40:6, and PC ae C44:4; and the sphingolipid (SM) (OH) C14:1 were detected in obese adolescents. In contrast, lower levels of several AC (C4, C9, and C14:1-OH), arginine, asparagine, glutamine, glycine, isoleucine, methionine, ornithine, serine and threonine, carnosine, dopamine, serotonin, PC aa C28:1, PC aa C30:2, PC aa C34:1, PC aa C34:2, PC aa C34:4, PC aa C36:1, PC ae C38:5, PC ae C38:6, PC ae C44:5, SM (OH) C22:1, SM C16:0 and SM C24:1 were observed in obese adolescents than in the non-obese controls.

Other authors have opted to perform targeted analysis using the same commercial kit with the advantage of providing comparable results (Table 1). For instance, Tulipani et al. (2016a, b) concluded that serum concentrations of lysoPCs (lysoPC a C17:0, lysoPC a C18:1, and lysoPC a C18:2) show a robust inverse correlation with BMI, body weight and waist and hip circumference in MO adults. Additionally, both choline- and ethanolamine-containing lysolipids acylated with margaric acid (C17:0) oleic acid (C18:1) and linoleic acid (C18:2) were the best classifiers of MO, together with diacyl and acyl-alkyl phosphocholines with LCFA (Tulipani et al. 2016a, b). Bagheri et al. (2018) reported that MHO phenotype was associated with alanine, tyrosine, glutamic acid, ACC18:2, acyl-lysoPC C18:1and c18:2, and alkyl-lysoPC C:18, and diacyl-phosphatidylcholines C312:1 and C38:3. Whereas the MUHO phenotype was associated to tyrosine, glutamic acid, serine, proline, and asparagine), AC C3:0, acyl-lysoPC C18:1, C18:2, and C16.1, diacyl-phosphatidylcholines C32:1 C32:2, C34:2, and C38:3, and acyl-alkyl-phosphatidylcholine C34:3. Both associations were detected when compared with healthy individuals; however, when both phenotypes were compared directly, there were no differences.

### Differences in response to dietary challenges between obese and non-obese individuals

Badoud et al. (2015b) studied the differences in the effect of a high-calorie meal administered in an acute intervention of 120 min among lean, healthy, MHO, and MUHO subjects (Table 2). Interestingly, the authors reported correlations between the levels of various BCAAs and FAs (e.g., saturated myristic and palmitic acids) with glucose levels and the insulin AUC. Moreover, several metabolites (asparagine, cystine, glutamine, serine, and the carnitine-to-acetylcarnitine ratio) exhibited different responses among the three groups. In addition, the serum concentrations of the PUFAs linoleic acid, γ-linolenic acid, and arachidonic acid showed subtle changes after the meal among the obese groups.

Geidenstam et al. (2014) initially studied the response of subjects with impaired glucose tolerance. Briefly, the authors reported three significant shifts to oral glucose tolerance test (OGTT), including compounds with a delayed glucose-provoked decrease (mostly FFAs), metabolites that showed a rapid onset (AAs and BCAAs) and compounds characterized by a blunted onset (Geidenstam et al. 2014). Second, in subjects who experienced weight loss and a weight maintenance period, changes in some but not all components of the OGTT-elicited serum profile that differed between obese glucose-intolerant subjects and lean glucose-tolerant subjects were observed (Geidenstam et al. 2014, 2016).

In a different type of challenge conducted by Baker et al. (2015), the authors studied the impact of a high-fat diet (HFD) administered for 5 days after profiling the obesity signature at baseline and revealed that muscle medium-chain AC (MCAC) (C6, C8, C10:2, C10:1, C10, and C12:1) levels are increased in obese subjects but decreased in lean subjects (Table 2). The plasma C10:1 content is also decreased in the lean subjects but increased in the obese subjects from pre- to post-HFD (Baker et al. 2015). Additionally, after the HFD intervention, lower glycine, histidine, methionine, and citrulline levels were observed in obese subjects. Moreover, skeletal muscle α-ketoglutarate levels were increased in the lean subjects from pre- to post-HFD conditions but were decreased in obese individuals (Baker et al. 2015). Furthermore, lean individuals exhibited decreases in MCAC (C4/4i, C6, C8, C10:2, C10:1 (also in plasma), C10, and C12:1) levels in response to the HFD, and the obese subjects showed increased levels from pre- to post-HFD. Both plasma C3 and C4/4i AC levels were elevated in the obese subjects compared with lean subjects.

Recently, a study focused on skeletal muscle was carried out in lean and obese men to explore and compare substrate metabolism in such tissue after fasting conditions (Table 2) (Bak et al. 2018). Interestingly, β-hydroxybutyrate was found elevated in plasma, being more pronounced in lean than obese. Also, plasma glycerol was increased in obese during 12 h of fasting, and ~ 50% more in lean than obese during 72 h of fasting.

### Metabolomics of weight loss

Three interventions included in this review focused on investigating potential predictor profiles/signatures of weight loss interventions.

According to Stroeve et al. (2016), 57% of the variation in weight loss success is predicted by baseline metabolic parameters. For males, the models were based on plasma lipid species (particularly sphingomyelins and phosphatidylcholines), whereas several AAs were included in the models for females, particularly in the models distinguishing subjects with obesity from subjects with MO (e.g., alanine, proline, and tyrosine were lower in women with obesity versus MO). The best predictive models were obtained for subjects with MO (including ketone bodies, triacylglycerols, phosphatidylcholines, AAs as valine, tyrosine, alanine, and proline, creatine and creatinine).

Geidenstam et al. (2017a) identified changes in the plasma levels of several AAs after weight loss for nine months and weight maintenance in obese subjects and evaluated in a replication cohort (Table 2). Of the 21 detected AAs, only tyrosine approached the lean reference profile (no initial increase) after weight loss, and this profile was maintained after weight maintenance (Geidenstam et al. 2017a).

Subjects enrolled in a 1-year weight loss program were classified into two groups according to the achievement (< or > 10% weight loss) (Geidenstam et al. 2017a). The analysis revealed that decreased levels of 1-methyladenosine, alanine, proline, trans-cinnamic acid, tyrosine, and the BCAAs were associated with a ≥ 10% weight loss. A lower baseline concentration of xylitol was predictive of a decrease in BMI and ≥ 10% weight loss.

Eight articles (Leal-Witt et al. 2018; Kang et al. 2018; Palau-Rodriguez et al. 2019; Mills et al. 2019; Almanza-Aguilera et al. 2018; Perez-Cornago et al. 2014; Zheng et al. 2016a, b) focused on determining the effect of weight loss on the metabolomics profiles of overweight and obese individuals. The article from Zheng et al. (2016b) was approached as two studies because the study design including two different cohorts, see below).

In prepubertal children with obesity, Leal-Witt et al. (2018) reported a decrease of urine trimethylamine N-oxide (TMAO) after following a Mediterranean diet with increased physical activity for 6 months (Table 2). Of the 32 distinct metabolites identified, highlighted metabolites altered after the intervention were xanthosine, 3-hydroxyisovalerate, and dimethylglycine (Leal-Witt et al. 2018).

In the shorter intervention, Perez-Cornago et al. (2014) studied the effect of an energy-restricted diet (Table 2). The concentrations of SFAs, including palmitic acid (C16:0) and stearic acid (C18:0), MUFAs (oleic acid (C18:1) and cis-11–eicosenoic acid (C20:1)), cis-11, 14-eicosadienoic acid (C20:2), and cis-4, 7, 10, 13, 16, 19-docosahexaenoic acid (C22:6n-3), and total, ω-6 and ω-3 PUFAs were significantly reduced, and isoleucine concentrations were decreased in serum after the intervention. Palmitoleic acid (C16:1) appeared to predict body fat loss negatively. Other short-term intervention showed significantly higher increases in plasma of MCAC and long-chain AC (LCAC) and lysoPC (20:4) in overweight adults with a low-calorie diet than their control group (Table 2) (Kang et al. 2018). A study including MHO women undergoing a lifestyle weight loss treatment for 3 months (and a 12-month follow-up) showed an altered plasma metabolome (Almanza-Aguilera et al. 2018). Namely, higher levels of formate and phosphocreatine and lower levels of trimethylamine were observed in the treatment group than in the control group. Moreover, higher myo-inositol, methylguanidine, and 3-hydroxybutyrate levels and lower proline levels were also detected in the treatment group; higher levels of hippurate and asparagine and lower levels of 2-hydroxybutyrate and creatine correlated with weight loss. Nevertheless, these changes were statistically significant after three months follow-up, but not at the 12-month follow-up. In another study, MHO women followed a hypocaloric Mediterranean diet with physical activity recommendations for 12 months (Table 2) (Palau-Rodriguez et al. 2019). The relative concentration of 1,5-anhydroglucitol was increased in plasma in the low weight loss (LWL) group after the intervention. The plasmalogen 1-(1-enyl-palmitoyl)-2-oleoyl-sn-glycero-3-phosphocholine (P-16:0/18:1) and the exogenous compound carotenediol were increased in the high weight loss (HWL) group, and significantly more so than in the LWL. Then, the levels of 3-(4-hydroxyphenyl) lactate and some sphingolipids (SM (d18:0/22:0) and SM (d18/0/20:0, d16:0/22:0)) decreased more after the intervention in the HWL versus LWL. Similarly, the androgens 16α-hydroxydehydroepiandrosterone 3-sulfate and androstenediol (3β, 17β) disulfate decreased in both weight loss categories, but a higher decline was observed in the HWL.

Using a different approach, Zheng et al. (2016a, b) analyzed urine, plasma, and fecal samples from individuals on an energy-restricted diet with either low or high-dairy intake for 24 weeks (Table 2). They reported increased levels of citrate, creatinine, and urea and decreased levels of hippurate and TMAO after a high-dairy intake compared with a low intake. Furthermore, the plasma metabolome reflected a change in the lipid and lipoprotein profile associated with the energy restriction. The changes in the fecal metabolome (mainly short-chain FAs (SCFAs)) were related to dairy intake. Furthermore, in a large cohort of pregnant women with obesity diet and physical activity intervention during pregnancy and up to 6 months lessened the reduction in the proportion of ω-6 and PUFAs and reductions in the rate of increase in the proportion of saturated FAs (Table 2) (Mills et al. 2019) Rates of increase in lactate, pyruvate, and alanine were reduced, and of acetate increased in comparison with the control group (Mills et al. 2019). Thereupon, the lifestyle intervention led to mitigating the metabolic changes during pregnancy that might be characteristic of obesity and pregnancy status.

Finally, Zheng et al. (2016b) analyzed data from the POUNDS LOST and the DIRECT studies (Table 2). Both cohorts followed a weight loss program with similar characteristics and were followed for 2 years, including a 6-month visit. The POUND LOST study revealed correlations between decreased nine plasma levels of AAs (BCAAs (leucine/isoleucine and valine), aromatic AAs (tyrosine and phenylalanine), and finally alanine, proline, sarcosine, hydroxyproline, and methionine) with weight loss; moreover, the DIRECT study validated the correlations with seven of these AAs (alanine, tyrosine, leucine/isoleucine, sarcosine, phenylalanine, hydroxyproline, and methionine). Additional analyses showed stronger correlations with these changes after 6 months, although the change persisted for 2 years.

Additionally, three interventions studied the effect of weight loss induced by physical activity. First, Meucci et al. (2017) reported that a four-week exercise program did not significantly change the metabolic signature of overweight preadolescents. Nevertheless, an eight-week program increased the urine concentrations of pantothenic acid, glyceric acid, l-ascorbic, xanthine, and adenosine compared to the control group (Meucci et al. 2017).Other intervention study assessed the effect of endurance training for 6 weeks on gut metagenome and plasma metabolites of overweight women (Table 2) (Munukka et al. 2018). The training intervention decreased plasma phospholipids and cholesterol in large VLDL particles, with not more significant alterations in any other plasma metabolites (including AAs, FAs, ketone bodies or gluconeogenesis-related metabolites) (Munukka et al. 2018). Likewise, no associations were studied between plasma metabolites and cardiovascular and inflammation biomarkers.

In a more extended intervention (Duft et al. 2017), three times/week for 24 weeks, including obese individuals in a combined training program, shifts in the serum levels of 20 metabolites were observed. Of these metabolites, tyrosine, 2-oxoisocaproate, histidine, and pyruvate appeared to be the best discriminators. Moreover, those metabolites were correlated with functional and biochemical parameters, such as strength, peak VO_2_, the percentages of fat mass and lean body mass, WC, and plasma insulin concentration.

### Dietary interventions focused on obesity and risk factors related to obesity

Four studies examined the effects of dietary interventions on obesity and obesity-related risk factors, such as inflammation and oxidative stress (Table 3). Nieman et al. studied the plasma and serum metabolomes of overweight women who ingested a red pepper spice supplement (Nieman et al. 2012a, b) or chia seeds (Nieman 2012b) for four and 10 weeks, respectively. The targeted analyses were focused on metabolites associated with inflammation and oxidative stress, but no significant changes were observed after the interventions.

Gu et al. (2013) studied the effect of a very low-carbohydrate diet (VLCD) on obese subjects (Table 3). The authors first identified the differences between obese and lean individuals. A serum profile comprised of increased levels of FAs, AAs, and carboxylic acids characterized the obese subjects. After consuming a VLCD for 8 weeks, the obese subjects exhibited several metabolic shifts in the levels of these metabolites. In parallel, specific alterations were also observed, including shifts in the arachidonate, cis-11, 14-eicosadienoate, cis-11, 14, 17-eicosatrienoate, 2-aminobutyrate, AC and threonate concentrations, all of which are involved in inflammation and oxidation processes.

Kim et al. (2013) administered a 12-week black soybean peptide dietary intervention and identified key metabolites associated with weight loss in healthy obese subjects (Table 3). After supplementation, the serum concentrations of metabolites such as betaine, benzoic acid, pyroglutamic acid, pipecolic acid, *N*-phenylacetamide, uric acid, l-aspartyl-l-phenylalanine, and lysoPCs (lysoPC a C18:1, lysoPC a C18:2, lysoPC a C20:1, and lysoPC a C20:4) were increased. Meanwhile, the levels of l-proline, valine, l-leucine/isoleucine, hypoxanthine, glutamine, l-methionine, phenylpyruvic acid, several carnitine derivatives, and lysoPCs (lysoPC a C14:0, lysoPC a C15:0, lysoPC a C16:0, lysoPC a C17:1, lysoPC a C18:0, and lysoPC a C22:0) were significantly decreased.

Recently, other authors have investigated the effect of Korean black raspberry (KBR) supplement for 4 weeks on overweight or obese individuals with a sedentary lifestyle (Kim et al. 2017) to identify metabolites that predict responses to such intervention against oxidative stress and inflammation (Table 3). Increased levels of several urinary AAs, organic acids and other type of metabolites were as betaine, N-phenylacetylglycine and phenylacetate were observed. Moreover, the levels of adenosine and carnitine decreased after the intervention. Authors concluded that higher level of glycine and *N*-phenylacetylglycine (as a two-metabolite set) had the most robust prognostic relevance for future interventions against oxidative stress.

Romo-Hualde et al. (2018) investigated the effects of eicosapentaenoic acid (EPA) and α-lipoic acid (α-LA) on urinary metabolomic profiles in overweight/obese women (Table 3).

The group supplemented with α-LA presented a weight loss that was associated with a highlighted presence of an ascorbate intermediate metabolite (one of the isomers of trihydroxy-dioxohexanoate, or dihydroxy–oxohexanedionate).

In a postprandial study, the metabolic profile from obese adults with MetS showed a differential response to low-fat milk or a rice beverage consumption (Table 3) (Xu et al. 2018). At 120 min, nine metabolites (i.e., orotate, leucine/isoleucine, mesoxalate, asparagine, citrulline, methionine, allantoin, ornithine, and tyrosine) were significantly altered in the low-fat milk versus the rice beverage group. The evaluation of a low-glycemic index diet in overweight/obese adults for 6 months (Table 3) (Hernández-Alonso et al. 2019) revealed several changes. The plasma serine levels were significantly increased following the low glycemic index diet compared to both the high glycemic index and low-fat diets. Tyrosine was decreased, and glycine was increased in the group receiving the low glycemic index diet versus that having the high glycemic index diet. Also, they observed a significant decrease in leucine and valine in the low glycemic index diet in contrast to the low-fat diet. Regarding lipids, several phosphatidylcholines (i.e., C32:1, C34:2e, C36:2e, C36:5e, C38:5 …) were significantly reduced after the low glycemic index diet versus the high glycemic index and/or low-fat diets.

Hibberd et al. (2019) conducted an RCT to study the effects of a probiotic (*Bifidobacterium animalis* subsp. *lactis* 420™) and/or a prebiotic (polydextrose) interventions in MHO subjects for 6 months (Table 3). Interestingly, the primary conjugated plasma bile acid, glycocholic acid, was reduced in the pre- and probiotic combination compared to placebo. In addition, secondary conjugated plasma bile acids were also reduced (Hibberd et al. 2019).

Finally, an intervention that contained pea fiber did not cause changes in the serum metabolites of overweight/obese adults after 12 weeks of consumption (Table 3) (Mayengbam et al. 2019). However, fecal SCFAs and bile acids were altered. For instance, acetate was significantly increased, and isovalerate decreased after pea fiber intervention (but not compared to placebo). The concentrations of fecal primary cholic acid and chenodeoxycholic acid, and secondary deoxycholic bile acid and total bile acids were significantly reduced in the intervention group.

## Discussion

The use of metabolomics to study obesity is increasing and improving our understanding of the alterations that occur during the development of obesity and their relationships with the disease. The results from the articles here included were categorized according to the different approaches reported. However, to facilitate the interpretation, the discussion will be focused on the metabolite classes to understand the different alterations related to obesity. Hence, metabolites are grouped in sexual steroids, AA and protein metabolism, AC, lipids, carbohydrates, and other relevant molecules (not included in a specific group).

### Metabolic features of obesity: characterization and importance of the metabolomics signature

Obesity is a whole-body adaptation to extra energy intake and decreased energy expenditure, mainly due to a sedentary lifestyle and a lack of physical activity. Also, obesity plays a crucial pathophysiological role in the development of IR, dyslipidemia and hypertension, leading to T2DM and an increased risk of CVD (Bastard et al. 2006; GBD 2015 Obesity Collaborators et al. 2017; Williams et al. 2015). Therefore, the characterization of the metabolomic signature in obese subjects might aid researchers in identifying those subjects at a higher risk of developing metabolic diseases, thus facilitating the timely administration of an appropriate treatment strategy.

#### Sexual steroids

The identification of a metabolic signature associated with age might provide more efficient preventive treatments for obesity before individuals reach adulthood. Body composition during puberty has been suggested to be a predictive marker of body composition in adulthood (Guo et al. 2002), as well as predicting the risk of comorbidities such as obesity, dyslipidemia and CVD (Baker et al. 2007; Vandewalle et al. 2015). Accordingly, both puberty and sex hormones have been shown to contribute to the development of obesity and CVD (Vandewalle et al. 2015; Widén et al. 2012; Zhai et al. 2015). For instance, Butte et al. (2015) detected an association between DHEA-S and BMI and adiposity in obese children. Moreover, prepubertal obese males showed significantly higher serum levels of DHEA-S, but also of testosterone than normal-weight children (Reinehr et al. 2005). However, Gawlik et al. (2016), did not identify any urinary steroid signature correlated with BMI. Nevertheless, we should highlight that these authors did not compare the obesity profile with lean controls.

Interestingly, the 16α-hydroxydehydroepiandrosterone 3-sulfate decreased after LWL, and even a more significant reduction was observed after HWL in MHO women (Palau-Rodriguez et al. 2019). These findings might reflect the modulation of endocrine metabolism due to weight loss. Accordingly, steroid sulfation and desulfation are fundamental pathways for endocrine balance, specifically for fat mass distribution and glucose metabolism (Mueller et al. 2015). Actually, DHEA-S is one of the most abundant steroids in human circulation and accumulate in AT at even higher concentrations (Bélanger et al. 2006). Furthermore, although the authors claimed that the steroid derivative, 12-ketoporrigenin, was also positively correlated with BMI (Zhao et al. 2016a, b), we should highlight that such a compound origin is related to the consumption of onion-family vegetables (Fattorusso et al. 2000) and such a finding should be carefully interpreted.

Nonetheless, the role of sexual steroids in obesity may differ at various life stages and with sexual dimorphism. For example, testosterone, the most critical androgen for males, has been described to be anti-adipogenic; its supplementation in adult men reduces abdominal fat by stimulating lipolysis and thereby reducing fat storage in adipocytes (Vitale et al. 2010). Even during pubertal development, lower testosterone concentrations have been observed in obese boys than in normal-weight boys (Mogri et al. 2013; Taneli et al. 2010). In addition, a consistent inverse correlation between testosterone levels and markers of adiposity in overweight adult males has been detected (Bann et al. 2015; Blouin et al. 2005; Gagnon et al. 2018; Gates et al. 2013; He et al. 2018). However, the data are less uniform in females, with no association (He et al. 2018) or a positive correlation observed in overweight-obese women (Bann et al. 2015; De Simone et al. 2001) and an inverse correlation observed in non-obese postmenopausal women (Casson et al. 2010). However, DHEA-S appears to play a more significant role in women body composition, both in young females (De Simone et al. 2001; Mäntyselkä et al. 2018) and in adult ones (Barrett-Connor and Ferrara 1996; De Pergola et al. 1994). The androgen receptor is expressed widely throughout the AT compartment, indicating that white AT adipocytes may be particularly sensitive to androgens (Newell-Fugate 2017). Hence, it is consistent with the findings reported by Palau-Rodriguez et al. (2019) in relation to the weight loss effect on steroid sulfates. Therefore, more studies focused on determining the roles of steroids in obesity are necessary, particularly from a metabolomics perspective.

#### AA and protein metabolism

One of the major groups of metabolites dysregulated in obesity is AAs, particularly BCAAs and AAAs. Phenylalanine concentrations were higher in obese individuals (Butte et al. 2015; Kim et al. 2010b; Fattuoni et al. 2018; Wang et al. 2018; Houttu et al. 2018; Yu et al. 2018). Moreover, tyrosine levels, a hydroxylation product of phenylalanine metabolism, have been associated with an increase in the hepatic fat content (Haufe et al. 2016), and its levels (as well as alanine) have been associated with prediction of successful weight loss (Stroeve et al. 2016) and weight loss per se following dietary and physical activity interventions (Duft et al. 2017; Geidenstam et al. 2017a; Zheng et al. 2016a, b). Furthermore, tyrosine contributes significantly to the profile defined in obese children and could serve as a posible predictor of IR in obese children (Hellmuth et al. 2016; Butte et al. 2015). In addition, p-cresol and p-cresol sulfate, degradation products of tyrosine and to some extent of the phenylalanine metabolism and phenylacetamide (an intermediate) were increased in plasma (Yu et al. 2018). Therefore, modifications in the phenylalanine and tyrosine metabolism might be a result of liver dysfunction associated with metabolic derangement (Libert et al. 2018). Further investigation is needed to determine if the study of the tyrosine metabolism could serve to identify the metabolic wellness of overweight and obese people.

Higher concentrations of tryptophan and its metabolites, kynurenine and kynurenic acid, have been detected in obese subjects and are associated with BMI (Yu et al. 2018; Ho et al. 2016; Zhao et al. 2016a, b; Carayol et al. 2017). Interestingly, alterations in the kynurenine pathway have been reported in subjects with obesity and IR (Favennec et al. 2015), and furthermore, increased levels of both metabolites have been detected in patients with diabetic retinopathy (Munipally et al. 2011). The higher levels of such compounds might reflect immune activation or low-grade systemic inflammation due to an increase in the enzyme indoleamine 2,3-dioxygenase (IDO) activity (Zhao et al. 2016a, b; Dadvar et al. 2018). The increased activity of IDO has been closely related to the propagation of obesity, probably, because the reduced tryptophan mediated by IDO may reduce serotonin production and cause mood disturbances, depression, and impaired satiety ultimately leading to increased caloric uptake and obesity (Brandacher et al. 2007). In contrast, decreased glycine levels have been detected urine, plasma and skeletal muscle samples from obese subjects and are inversely correlated with BMI (Baker et al. 2015; Butte et al. 2015; Cho et al. 2017; Kraus et al. 2016; Newgard et al. 2009). Although little is known about the pathophysiological mechanisms associated with glycine depletion, glycine utilization in patients with diabetes is increased because of excess acyl group formation (Adeva-Andany et al. 2018).

Several studies in animal models support a close association between obesity state and plasma (She et al. 2007; Sailer et al. 2013) and urine citrulline levels (Connor et al. 2010), indicating impairment of the hepatic amino acid handling. A possible hypothesis suggested is regarding the changes of AAs involved in the urea cycle in obesity, that could indicate in turn an alteration of urea synthesis in the liver, i.e., a block in the cytosolic reactions with increased ornithine and citrulline levels (She et al. 2007) and therefore a reduced systemic arginine bioavailability (decreased ratio of plasma arginine to ornithine + citrulline) (Tang et al. 2009; Sailer et al. 2013). HFD mice presented a reduction of arginine levels, while citrulline levels were elevated (Sailer et al. 2013). However, metabolomics findings in human studies have demonstrated the opposite. For instance, an inverse association of human plasma citrulline with BMI was observed by Ho et al. (2016) and lower citrulline concentrations were observed in the skeletal muscle of obese subjects compared with lean controls (Baker et al. 2015). Furthermore, the citrulline levels (along with other AAs such as leucine/isoleucine, mesoxalate, asparagine, methionine, allantoin, ornithine, and tyrosine) were higher at postprandial measurement in the low-fat trial compared to the rice milk in obese population with MetS, probably because of the higher protein content of low-fat milk (Xu et al. 2018). Although unaltered levels of serum citrulline in obese subjects have been reported (Newgard et al. 2009), the most common finding is to encounter this compound decreased in obese subjects and in patients with diabetes (Park et al. 2015). Nevertheless, the physiological cause that might explain this inverse association is still unknown, although it might be related to the degree of liver steatosis, which is usually present in the obese state.

Uric acid levels are substantially increased in obese subjects and proportionally associated with BMI (Park et al. 2015; Ho et al. 2016; Ruebel et al. 2019) and recently associated with BMI (Cirulli et al. 2019). Additionally, it has been revealed that hyperuricemia is a predictor of IR and T2D debut (Gil-Campos et al. 2009; Krishnan et al. 2012). Hyperuricemia may also cause obesity by accelerating hepatic and peripheral lipogenesis (Johnson et al. 2011) and could also reflect high oxidative stress as it is known as an antioxidant and scavenger of free radicals.

On the other hand, the characteristic increased exogenous consumption of proteins and therefore endogenous production of uric acid due to the purine catabolism in the obese population are additional factors that result in hyperuricemia (Remedios et al. 2012). Hence, the evidence for increased uric acid levels in obese individuals is widely known, although its change in response to dietary interventions should be studied with a more exhaustive design and statistical adjustments; Kim et al. (2013) reported increased uric acid levels after the administration of the black soybean peptide supplement to overweight/obese subjects with a subsequent weight and body fat loss. This unexpected finding was not discussed and probably the change of dietary habits because of the soybean intervention might influence on the unadjusted data for protein consumption.

Regarding BCAAs, associations between elevated serum concentrations with obesity, IR, and other complications were observed several decades ago (Felig et al. 1969; Newgard et al. 2009). In fact, many studies have described higher blood levels of BCAAs both in obese children (Butte et al. 2015) and adults (Kim et al. 2010b; Newgard et al. 2009; Xie et al. 2014; Wang et al. 2018) and positive correlations with anthropometric markers and fat mass (Foerster et al. 2015). Stroeve et al. (2016) detected higher concentrations of BCAAs in MO women than in obese women. By contrary, decreased levels of BCAAs have been associated with weight loss in obese subjects (Geidenstam et al. 2017a), after weight loss through diet (Kim et al. 2013; Zheng et al. 2016b; Hernández-Alonso et al. 2019) or physical activity in pregnant women (Mills et al. 2019). The increase, or decrease during weight loss, of BCAAs, reflect the status of the protein breakdown, which is a consequence of IR, thus pointing towards to metabolic complications. In fact, the higher concentrations of glutamate observed in six studies (Butte et al. 2015; Newgard et al. 2009; Yu et al. 2018; Maltais-Payette et al. 2018; Wang et al. 2018; Carayol et al. 2017) might be linked to the BCAAs alterations, since such an AA is produced as the first step of BCAAs catabolism (Newgard 2017).

Elevated concentrations of ADMA in obese subjects (Cho et al. 2017; Feldman et al. 2019; Butte et al. 2015) is associated with endothelial dysfunction, most probably due to the reduction of arginine availability (Eid et al. 2004; El Assar et al. 2016). Interestingly, such findings are reported in adolescents and adult subjects, and it would be of keen interest to investigate in-depth if this compound might serve as a marker of severity of endothelial dysfunction.

Furthermore, specific intermediate metabolites of AAs have been reported to be important markers of obesity and its complications (Newgard et al. 2009). For instance, lower levels of α-KIC, a metabolite derived from leucine, were detected in the subcutaneous AT of obese subjects (Hanzu et al. 2014), along with diminished leucine uptake in the obese visceral fat depots. The dysregulation of leucine metabolism seems to be increased in the visceral obese AT, preventing the formation of α-KIC. Moreover, catabolic pathways of leucine through the KIC acid route involve the formation of substantial amounts of alanine and glutamine. These pathways are the route for the disposal of amino groups released from the transamination of BCAAs (Newgard et al. 2009). Therefore, as alanine and glutamine are highly gluconeogenic AAs, a possible hypothesis is that the increased amount of alanine released by the visceral AT to the systemic circulation contributes to hyperinsulinemia and the development of IR.

#### Acylcarnitines

AC are organic compounds containing an FA, with the carboxylic acid attached to carnitine through an ester bond. The roles of fatty AC species reflect different situations in the organism. Increased levels of SCAC species indicate amino acid anaplerosis; medium-chain species reflect distal β-oxidation (i.e., downstream of CPT-1) whereas long-chain species reflect transport and proximal β-oxidation efficiency (i.e., including an upstream of CPT-1). The sentinel species C6, C8, C10, and C10:1 have been used to evaluate the MCAC flux through the β-oxidation pathway (Baker et al. 2015). Hence, AC might serve as relevant biomarkers of IR and are defined as a by-product of fat and amino acid oxidation in mitochondria (Makrecka-Kuka et al. 2017). Obese subjects have shown a high rate of incomplete FA oxidation, abnormal AC profiles, and AAs biosynthesis, along with the perturbation of mitochondrial metabolites (Schooneman et al. 2013). In obese children, the levels of several SCAC, such as C5-OH, C3, C4 (Butte et al. 2015), C12:1 and C16:1 (Wahl et al. 2012), are elevated. A higher C3 level was also detected in obese men (Baker et al. 2015; Xie et al. 2014; Piening et al. 2018), as well as higher C4/4i, C5, and C5:1 levels (Baker et al. 2015; Cirulli et al. 2019; Piening et al. 2018). Moreover, obese subjects exhibit increased concentrations of C4/4i, C6, C8, C10:1, and C10:2 in skeletal muscle AC, and C3 and C4/4i in plasma in response to the HFD challenge compared with lean subjects (Baker et al. 2015). Overall, the observed accumulation of BCAAs in the flux increases its catabolism in the liver and skeletal muscle. As a result, the elevated concentration of BCAAs may hypothetically be associated with the higher concentrations of the SCACs C3 and C5 as C3 AC reflects the propionyl CoA pool; propionyl CoA is a by-product of both isoleucine, and valine catabolism and C5 AC are comprised of α-methylbutyryl and isovalerylcarnitine species; α- methylbutyryl CoA and isovaleryl CoA are intermediates in mitochondrial isoleucine, and leucine catabolism, respectively, and these intermediates equilibrate with their cognate AC esters (Newgard et al. 2009; Schooneman et al. 2013; Feldman et al. 2019). Whereas C4 can be produced in both amino acid and fatty acid catabolism (Koves et al. 2008).

Remarkably, Kang et al. (2018) observed increases in plasma MCAC AND LCAC in overweight adults with a low-calorie diet and these changes were negatively correlated with changes in visceral fat areas (Kang et al. 2018). Such findings could be explained as increased oxidation of the free FAs (FFA) released from the visceral fat due to the weight loss, thus generating MCAC and LCAC. Then, the increase of MCAC and LCAC levels may be driven by an improvement of the acetyltransferase activity rather than an unbalanced mitochondrial fatty acid oxidation (Schooneman et al. 2016; Kang et al. 2018).

#### Lipid metabolism

Lipids have diverse roles as signaling molecules, metabolic substrates, and cellular membrane components. The chain length and the degree of desaturation of the FA moieties in lipid molecules increase the complexity of biological roles assigned to various lipid classes. Moreover, lipids that are synthesized endogenously or obtained through diet exhibit differences in accumulation and/or metabolism and subsequent biological roles (Yang et al. 2018).

In the current study, we reported diverse findings from various studies, namely, changes in FAs, such as PUFAs and SFAs, as well as more complex lipids, such as sentinel lysophospholipids and sphingomyelins.

Houttu et al. (2018) identified a lipid signature in obese pregnant women characterized by high VLDL subclasses and lower HDL particles and other PUFAs. Furthermore, according to Mills et al. (2019), obese pregnant women following a lifestyle intervention reduced their ω-6 and total PUFAs. Such a profile should be related to lifestyle and diet rather than a consequence of obesity. Remarkably, the study of the placenta from obese women revealed a lipid profile that suggested a disruption of the LCPUFA biomagnification that might impact in the risk of adverse fetal outcomes and of the development of metabolic diseases throughout postnatal life (Fattuoni et al. 2018). In fact, this is corroborated in the study from Sorrow et al. (2019) in which the presence of elevated lipid species, including linoleate, myristate, oleate, palmitate, stearate, caprate, and species of AC in the umbilical cord was associated with the development of obesity at 3 to 5 years of age (Sorrow et al. 2019).

The serum concentrations of SFAs, including C16:0 and C18:0, and MUFAs (C18:1 and C20:1), and total, ω-6, and ω-3 PUFAs were decreased after the weight loss intervention in the study by Perez-Cornago et al. (2014). Moreover, C16:1 was suggested to serve as a negative predictor of body fat loss. Concretely, SFAs are positively correlated with the development of obesity and diabetes, increasing complications related to metabolic disease (Jakobsen et al. 2009; Kien et al. 2013).

Controversial findings related to lysoPC have been reported. Some authors have reported decreased levels of these compounds (Barber et al. 2012; Heimerl et al. 2014; Cirulli et al. 2019), whereas other evidence suggests a correlation between plasma levels of lysoPC, sphingomyelins and phosphatidylcholines with obesity, although the pathways are not yet completely understood (Rauschert et al. 2016) but is hypothesized that such perturbations are a consequence of changes in weight rather than being a contributing factor.

Furthermore, phospholipids are also a group that characterizes the obesity profile. Indeed, obese mice treated with PC 18:0/18:1 (1-Octadecanoyl-2-(9Z)-octadecenoyl-sn-glycero-3-phosphocholine) exhibited increased glucose tolerance and IS (Liu et al. 2013). Numerous studies reported in the current review have described profiles of several phospholipids. For instance, Piening et al. (2018) detected a signature associated with BMI, which included several lysophospholipids and that respond to fluctuations in the BMI. This was suggested to be related to a decrease in the catabolism due to increased caloric intake.

Higher lysoPC a C18:0 concentrations have been identified in urine samples from obese adolescents (Cho et al. 2017) and in plasma samples from overweight/obese men (Kim et al. 2010b). Interestingly, after a black soybean peptide intervention for 12 weeks, the levels of this lysoPC species were decreased (Kim et al. 2013) and MHO showed lower levels than MUHO subjects (Bagheri et al. 2018). A similar phenomenon was detected for lysoPC a C14:0 in the same dietary intervention study (Kim et al. 2013).

Moreover, the lifestyle intervention by Mills et al. (2019) in obese pregnant women, resulted in a decrement in the rate of increase of phospholipids along with triacylglycerols in extremely large, very large, large and medium VLDL particles (Mills et al. 2019).

In contrast, decreased lysoPC a C18:1 and C18:2 were detected in obese children and adults (Wahl et al. 2012; Kim et al. 2010b), and both compounds were inversely associated with BMI (Bagheri et al. 2018). Whereas, an increase was observed after a black soybean intake (Kim et al. 2013). Moreover, significantly lower plasma lysoPC a C18:2 levels were observed in MO men and women than in obese subjects (Stroeve et al. 2016). In a young population, the PC a C34:1 and C34:2 levels are decreased in obese individuals (Cho et al. 2017; Wahl et al. 2012). While lower PC a 20:4 levels were only observed in obese children (Wahl et al. 2012). Nevertheless, the levels of these three lipids increased in obese adults after the black soybean peptide intervention (Kim et al. 2013). Overall, the presented evidence points towards an association of phospholipids with a diet mainly high-fat composed and might be regulated through the addition of fiber-contained products.

Differences in the levels of other phosphatidylcholines, such as PC a C34:4, PC a C38:5, and PC a C38:6, sphingomyelins and hydroxysphingomyelins, such as SM C16:0 or SM (OH) C22:1, PC ae (C38:7), PC ae (C40:6), PC ae (C38:3), and PC ae (C40:4) between healthy and obese subjects have been observed in some studies, but not in other studies, and thus these molecules are not considered critical metabolites in obesity signatures or as markers of weight loss.

Overall, the role of these lipid species remains to be investigated in-depth in humans. Nevertheless, some findings are consistent and might at least partially explain the physiopathology of obesity and the associated everyday habits, such as high-fat intake. For instance, higher saturated fat intake with a lower ratio of PUFAs/SFAs and relatively lower carbohydrate levels in overweight/obese than in lean subjects might partially explain the higher levels of lysoPC a C18:0 (Kim et al. 2010b, 2014). The hypothesis that the FA composition of serum (lyso-) phospholipids partially reflects an individual’s medium-term dietary FA intake has already been supported (Hodge et al. 2007).

#### Carbohydrates

Carbohydrate metabolism is vital for all metabolic processes, and its roles in the development and maintenance of obesity have been a matter of debate for decades. Glucose is catabolized via glycolysis to pyruvate, which is converted into acetyl coenzyme A (CoA), the entry point into the TCA cycle, under aerobic conditions (Park et al. 2015). Xylitol may be used as a substrate in the pentose phosphate pathway to produce fructose-6-phosphate that can generate acetyl CoA, a primary substrate for the TCA cycle, via glycolysis. Most likely, because of its implication in the TCA cycle, xylitol has been investigated in some studies. A lower baseline concentration of xylitol predicted a more significant decrease in BMI and ≥ 10% weight loss in subjects after a 1-year weight loss program (Geidenstam et al. 2017a). Human metabolism of xylitol, as well as its absorption from food (Islam and Indrajit 2012) or potential involvement of the gut microbiota, is not well understood. A study in mice detected significant changes in the microbiota following daily dietary supplementation with xylitol; the abundance of the phylum *Firmicutes* was increased in the group fed an HFD with xylitol solution (Park et al. 2015). The phylum *Firmicutes* accelerates the degradation of food components to supply energy to the host, and therefore is considered an obesity-related bacterial phylum (Khan et al. 2016).

Interestingly, 1,5 anhydroglucitol, a monosaccharide found almost in all foods, has been proposed as a biomarker of short-term glycemic control, for screening undetected T2D in saliva (Mook-Kanamori et al. 2014) and associations with BMI and adiposity indicators have been shown (Lipsky et al. 2016). In fact, a reduction after a lifestyle intervention in women and reported positive associations of such a compound with weight variables has been found (Palau-Rodriguez et al. 2019).

Mannose is one of the most common glucose metabolites reported in the current SR at incremented levels, both in obese adults (Fiehn et al. 2010; Gogna et al. 2015; Moore et al. 2013; Park et al. 2015) and in obese children (Butte et al. 2015). The liver is the main organ for mannose consumption; thus, an abnormal utilization will reflect higher concentrations in plasma. It is hypothesized that mannose may play a role in the development of IR, as it will reflect defective glycosylation that could also affect the insulin receptors in the liver tissue (Lee et al. 2016).

In this context, targeted metabolomics research that includes an analysis of glucose metabolites in obese and lean individuals will provide comprehensive information about their contributions to the metabolic signature of obesity and facilitate a determination of whether these metabolites might be targets for obesity treatments.

#### Other relevant molecules

Other molecules reported in the literature have controversial roles, such as the nucleoside, adenosine. Although no evidence has suggested a possible correlation between the levels of this nucleoside and the obesity signature, polyphenol supplementation in overweight or obese adults decreases the plasma adenosine levels (Kim et al. 2017). In contrast, a physical activity program increased urinary adenosine concentrations in overweight preadolescents (Meucci et al. 2017). The role of adenosine in obesity is not clear, since it not only participates in the obesity but is also involved in the initiation of obesity, and it may have anti-obesity activities as well (Pardo et al. 2017). Adenosine promotes adipogenesis by activating the A1 receptor and inhibits adipogenesis mediated by the activation of the A2B receptor in preadipocytes (Gharibi et al. 2012). In this context, adenosine exerts a receptor- and tissue-dependent effect. For example, adenosine receptor activation impairs insulin action in skeletal muscle (Pardo et al. 2017).

2-Ketoglutarate, also known as α-ketoglutarate or 2-oxoglutarate, is a key intermediate metabolite of one of the most fundamental biochemical pathways in carbon metabolism, the TCA. According to a study in mice, the administration of α-ketoglutarate might affect body weight and innate intestinal immunity by influencing the intestinal microbiota (Chen et al. 2017). Also, α-ketoglutarate has been associated with the induction of skeletal muscle hypertrophy and inhibition of protein degradation. In obese subjects, urine and skeletal muscle were decreased (Newgard et al. 2009; Baker et al. 2015), despite a targeted approach revealed positive correlations with BMI abdominal obesity, HOMA-IR and triacylglycerol levels (Ho et al. 2016). Therefore, further studies should focus on the role of this compound and its relationships with obesity, protein degradation, hypotrophy, and the intestinal microbiota.

In urine, serotonin levels were observed lower in young obese than normal-weight subjects (Cho et al. 2017). In the central nervous system, serotonin is intricately involved in appetite and subsequent nutrient intake (Tecott 2007), primarily regulated by processes innervated in the hypothalamus (Yabut et al. 2019). In fact, the inhibitory effect of serotonin on appetite has led to the approval of receptor agonists for the treatment of obesity (Bohula et al. 2018; Fidler et al. 2011; O’Neil et al. 2012; Smith et al. 2010) or even treatments based on the serotonin precursor 5-HTP that are involved in the meal satiation and the end state of post-meal satiety (Halford et al. 2005).

TMAO has been reported as biomarker of obesity (Zheng et al. 2016a, b), CVD risk (Bennett et al. 2013; Trøseid et al. 2015) and it is originated from microbial activity (Leal-Witt et al. 2018; Zheng et al. 2016a, b). Its precursor trimethylamine (TMA) provided from the microbial metabolism of dietary carnitine and choline, decreased in weight loss conditions (Almanza-Aguilera et al. 2018). TMA is oxidized by hepatic flavin-containing monooxygenases to form TMAO, which has been shown to be both proatherogenic and associated with CVD (Tang et al. 2013; Wang et al. 2011). Almanza-Aguilera et al. (2018) reported that lower levels of TMA after weight loss associated to lifestyle intervention are related to either a lower intake of its dietary precursors (i.e., eggs and meat) (Koeth et al. 2013; Tang et al. 2013) or modulation of choline and carnitine metabolism. Similarly, TMAO was reduced in urine after a lifestyle intervention program in obese prepubertal children (Leal-Witt et al. 2018). Although cholesterol levels in these children were within the average values, the authors detected a positive association between the changes in TMAO and cholesterol levels. Additionally, TMAO decreases expression of two key enzymes, CYP7A1 and CYP27A1, essential for bile acid biosynthesis and multiple bile acid transporters (OATP1, OATP4, MRP2, and NTCP) in the liver, which decreases bile acid pool, resulting in decreased reverse cholesterol efflux (Koeth et al. 2013). In this context, the primary conjugated plasma bile acid, glycocholic acid along with secondary conjugated plasma bile acids (glycoursodeoxycholic acid, taurohyodeoxycholic acid, and tauroursodeoxycholic acid) were reduced in the pre- and probiotic combination compared to placebo after 6 months (Hibberd et al. 2019). In feces, the primary bile acids, cholic acid, and chenodeoxycholic acid, the secondary deoxycholic acid and overall total bile acids were reduced after 12 weeks of pea fiber consumption (Mayengbam et al. 2019). It is known that bile acids are synthesized from cholesterol and excreted through the feces (Ma and Patti 2014). Thereupon, as fiber (especially insoluble fiber) increases fecal mass, a dilution of bile acids content might be expected (Woodbury and Kern 1971).

### Obesity as a risk factor for other metabolic comorbidities

Obesity is a risk factor for the development of several metabolic disorders. In fact, many authors reported metabolic alterations, such as IR, related to obesity or weight. For instance, decreased decrease in the uptake of BCAAs in obese subjects (21) might explain the accumulation of these metabolites in the bloodstream and the subsequent progressive obesity-associated complications. Hence, elevated BCAA concentrations may also serve as a biomarker for an increased risk of metabolic syndrome (Iwasa et al. 2015; Newgard et al. 2009; Shah et al. 2010). For example, Badoud et al. (2015b) reported correlations between the levels of BCAAs (and FAs) with glucose levels and insulin AUC, Xie et al. (Xie et al. 2014) reported a correlation with IR and Haufe et al. (2016) reported a correlation with hepatic IR. Furthermore, negative correlations between whole-body IS (calculated using the composite insulin-sensitivity index (C-ISI)) with plasma BCAA concentrations have been reported (Haufe et al. 2016). Similarly, Tulipani et al. (2016a, b) reported a positive correlation between valine levels and the degree of IR, independent from the BMI. Glutamate levels showed positive correlations with fasting insulin levels and the HOMA-IR index, while glycine concentrations were negatively correlated with the same parameters.

Ketone bodies, such as α-hydroxybutyrate, are increased in obese children plasma (Butte et al. 2015) and have been identified as strongly associated with the obese and diabetic state (Fiehn et al. 2010; Gall et al. 2010; Stroeve et al. 2016). Interestingly, α-hydroxybutyrate has been investigated as an early marker of both IR and impaired glucose regulation in a nondiabetic population (Gall et al. 2010). Furthermore, the ketone body β-hydroxybutyrate was highly elevated in the skeletal muscle of obese men (more than in lean) during prolonged fasting (up to 72 h) (Bak et al. 2018). The greater increment of ketone bodies in obesity status might be the result of the increased catabolism of more available BCAAs, but increased metabolization of intracellular skeletal fat mass could also contribute to the increased production of ketone bodies. Hence, all the findings regarding the implications of several metabolites on IR progression in obese subjects might elucidate the relevance of metabolomics to explain the obesity-associated complications.

A hypothesis proposed to explain the development of IR in obesity is focused on the lipotoxicity. This hypothesis states that an oversupply of fats that exceed the capacity of adipocytes leads to storage in other tissues. Consequently, these cells produce bioactive lipids that reduce IS and fat flow into the cell (Rauschert et al. 2016). LysoPCs are derived from PCs during LDL oxidation via either the lecithin-cholesterol acyltransferase (LCAT) or the lipoprotein-associated phospholipase A2 (LpPLA2) pathway. In fact, LpPLA2 activity has been reported to be increased in obese children. As a significant component of oxidized LDL, saturated lysoPCs exert pro-atherogenic and pro-inflammatory effects and impair insulin signaling (Murugesan 2003; Wahl et al. 2012). Consistent with these findings, Rauschert et al. (2016). observed a significant correlation between increased lysoPC a C14:0 levels with a high HOMA-IR in normal-weight young adults.

Thus, the current controversy regarding the causative role of and mechanisms underlying the effects of specific lipid classes on the subsequent development of IR in obese subjects is not surprising. However, new high-resolution metabolomics techniques have enabled the identification of lipid subclasses, and novel families of lipids that might even regulate IS, such as FA esters of hydroxy FAs (FAHFAs), diacylglycerols (DAG) and ceramides (Yang et al. 2018).

### Metabolic challenges

The knowledge acquired through metabolomics in metabolic challenges is fundamental due to the real-time data obtained that reflects the dynamics of the human metabolome. A standard definition of metabolomics is that it provides “a snapshot” of the metabolism; however, in some situations, such as in patients with a particular disease, researchers must understand the dynamics and obtain a “motion picture” of the events occurring in the metabolism. For example, Geidenstam et al. (2014) first studied the differential response of lean and obese subjects to an OGTT and reported evident disruptions in the regulation of ketogenesis, lipolysis, and proteolysis in the obese individuals. Afterward, the aim was to study the response of an overweight/obese population to an OGTT after weight loss and a weight maintenance period. As expected, the response differed between obese glucose-intolerant individuals and lean glucose-tolerant subjects (Geidenstam et al. 2014). Most likely, the most exciting result is that those changes occurred in a temporally different manner that coincides with improvements in either hepatic or peripheral IS during weight loss and weight maintenance, respectively (Geidenstam et al. 2017a).

Badoud et al. (2015b) concluded that FAs, such as 14:0 and 16:0 should serve as distinct markers of fasting and/or postprandial IS whereas SFA 18:0 can be inversely related to the fasting glucose levels. Interestingly, shorter chain SFAs were previously linked with an unhealthy cardiometabolic profile compared to longer chain SFAs (18:0, 22:0 and 24:0) (Badoud et al. 2015b). Subsequently, 18:0 SFA and ω-6 PUFA were inversely correlated with fasting glucose levels in adults, regardless of the BMI (Badoud et al. 2015b). Furthermore, MHO individuals showed better adaptability to the caloric challenge when compared to the MUHO individuals as the former preserved the IS. These findings are relevant if replicated in larger cohorts, in the translation to potential diagnostic use. We should note that recently, Bagheri et al. (2018) could identify differences between MHO and MUHO phenotypes when compared with normal-weight subjects but not when compared among them. Although their analysis had broad coverage, their reported metabolic signatures did not include any of the compounds reported by Badoud et al. (2015b).

Moreover, Baker et al. (2015) performed a 5-day HFD intervention that altered the metabolism of AC species, and its relationship with limited β-oxidation has already been discussed.

Bak et al. (2018) detected FFAs (palmitate, stearate, and arachidate) lower during 12 h of fasting in obese versus lean subjects. In addition, free carnitine that sustains the FFAs transportation into the mitochondria was lower in obese than lean participants. This phenomenon could indicate that there is a lower rate of release of FFAs during fasting in obese subjects that even might facilitate the insulin response at short-term in such population. Nevertheless, the regulatory mechanism of FFAs in context with insulin actions requires more detailed studies and to consider cautiously the processes of fasting condition that could result controversial in humans.

### Future perspectives

As shown in the present review, metabolomics studies facilitate the identification of metabolites involved in obesity by observing variations in metabolite concentrations in obese/overweight subjects compared with healthy individuals. Additionally, metabolomics has been used to discover biomarkers for several clinical conditions (Vinayavekhin et al. 2010). Biomarkers are regularly used in clinical practice to measure disease severity and provide essential prognostic information related to survival (Park et al. 2015). Using metabolomics studies, the clinical practice and the studies of obese subjects might be more productive and focused on specific metabolites and critical pathways to treat or even prevent the development of obesity and its severe complications. Furthermore, an understanding of the metabolic signature of obesity and its dynamics should lead to elaborate subclassifications within obese patients, according to their metabolic characteristics. These profiles would help clinicians to either screen individuals or identify and characterize outliers in clinical trials designed to test solutions for obesity. In this regard, a very comprehensive guide for helping in the developing of future nutrimetabolomics studies is presented by Ulaszewska et al. (2019) and must serve to harmonize the field.

Significant findings are presented in this review and suggest that several tracks should be followed. Moreover, more targeted and well-designed studies should focus on compounds such as BCAAs, AC species, and phospholipids. Their pathways must be delineated, and in-depth studies of not only the metabolites but also the catabolites and cometabolites should be performed to understand their relevance.

Currently, the study of the microbiome is gaining importance, and researchers have hypothesized a link between obesity and the microbiome. Nevertheless, an integrative approach, including metabolomics, might improve our understanding that not only the variability but also the function of the microbiome may lead to dysregulations in obesity, and further research must be developed. This approach will help researchers to clarify and understand the interactions of the microbial metabolites with the host organism and to avoid misinterpretations when reporting allegedly dysregulated compounds that might be related to the microbiome or to the diet. For instance, metabolites such as hippurate, isobutyryl glycine, and isovaleryl glycine, and TMAO were reported as biomarkers of obesity and might originate from microbial activity (Zheng et al. 2016a, b; Almanza-Aguilera et al. 2018).

The field still faces many challenges. During the elaboration of this review, we encountered many problems with the interpretation of the results due to the lack of a unified reporting scheme. Based on the metabolomics standards initiative (MSI) and core information for metabolomics reporting (CIMR) (Creek et al. 2014; Salek et al. 2015; Sumner et al. 2007, 2014), these deficiencies must be improved, and this topic has been reviewed elsewhere (Considine et al. 2018). Furthermore, although a consensus regarding the metabolites that comprise the metabolic signature of obesity has been achieved, further studies are needed to ensure the homologation, proper identification and validation of these features to guarantee their reliability.

In the present study, we have used the quality assessment tool QUADOMICS and, although it has been adapted for omics studies, we found some deficiencies in metabolomics reporting. Our major concern is related to the evaluation of the reporting of identification. This topic should be covered in-depth to provide the reader with a concise view of the level of identification of the features reported and thus, integrating these criteria might strengthen the reliability of the findings. For instance, a correct identification will provide tools to discriminate between endogenous and exogenous metabolites and reduce misinterpretations correctly. Therefore, researchers should develop a specific tool for assessing the quality of metabolomics studies. As a final reflection, the combined use of untargeted and targeted approaches should be acknowledged as complementary. Untargeted metabolomics is the best approach to generate a hypothesis or to detect compounds that were not initially contemplated or were utterly unknown, thus generating new knowledge. Nevertheless, several drawbacks must be considered when using untargeted approaches to advance the field, namely, the validation of protocols, workflows and standards, the consolidation of the features reported with a rational software (including the correct grouping of adducts) and the correct use of databases. In addition, targeted studies are necessary to validate and quantify the changes in metabolism in a more precise manner.

## Conclusions

Metabolomics provides a better understanding of disease progression and metabolic pathways in obese subjects. The present SR provides valuable information on specific metabolite patterns as characteristics of obesity, such as the metabolically healthy and unhealthy phenotypes, and even possible metabolomic profiles associated with their complications. These metabolites can be considered as biomarkers of obesity and improve our understanding of disease progression and metabolic pathways. Nevertheless, significant progress is needed, and further studies are required to test whether the proposed metabolites are considered an established and specific metabolic signature. If this goal is accomplished, the signature might be useful as a clinical tool and for the development of more accurate clinical treatments focused on the pathogenesis of obesity and its potential comorbidities.

## Electronic supplementary material

Below is the link to the electronic supplementary material.

Supplementary material 1 (XLSX 21 kb)
